# Prevalence and Mitigation of Cardiovascular Disease Risk Factors Among the Corporate Workforce in Sub-Saharan Africa: A Systematic Review and Meta-Analysis

**DOI:** 10.7759/cureus.75888

**Published:** 2024-12-17

**Authors:** Abiodun Bamidele Adelowo, Nestor Lemos Ferreira, Animesh Gupta, Zahid Khan

**Affiliations:** 1 Cardiology, University of South Wales, Wales, GBR; 2 Acute Internal Medicine, Southend University Hospital NHS Trust, Southend on Sea, GBR; 3 Acute Internal Medicine/Intensive care, Barking, Havering and Redbridge Hospital NHS Trust, London, GBR; 4 Acute Medicine, Mid and South Essex NHS Foundation Trust, Southend on Sea, GBR; 5 Cardiology, Bart’s Heart Centre, London, GBR; 6 Cardiology and General Medicine, Barking, Havering and Redbridge University Hospitals NHS Trust, London, GBR; 7 Cardiology, Royal Free Hospital, London, GBR

**Keywords:** behavioural risk factors, " "cardiovascular disease risk factors, corporate workers, corporate workplace, intermediate risk factors, pico (population, preferred reporting items for systematic reviews and meta-analyses(prisma), risk of cardiovascular diseases, sub-saharan africa, workplace wellness program

## Abstract

Cardiovascular disease (CVDs) is the leading cause of mortality worldwide. Corporate workplaces have been identified as important environmental factors that can increase the risk and severity of CVDs. Evidence indicates that the risk and severity of CVDs can be effectively reduced by mitigating modifiable behavioural and intermediate risk factors. Although the prevalence of CVDs and their associated risk factors is increasing in sub-Saharan Africa (SSA), most published data from the region are hospital-based and may not be true estimates. This study investigated the prevalence and distribution of CVD risk factors among the corporate workforce in SSA and the effects of workplace wellness programmes (WWP) on these risk factors. Accordingly, a systematic search was performed using Google Scholar, Cochrane Library, PubMed, MEDLINE, Scopus and Science Direct for articles published between January 2010 and March 2024. A total of 105 studies (*n* = 76,027) across nine countries met the eligibility criteria and were analysed. The pooled prevalence of the risk factors was unhealthy diet (80%), high salt intake (32%), stress (58%), poor sleep (59%), physical inactivity (PI, 59%), alcohol consumption (29%), harmful alcohol consumption (26%), tobacco smoking (7%), khat chewing (6%), overweight (36%), obesity (23%), central obesity (44%), high blood pressure (29%), high total cholesterol (33%), high low-density lipoprotein cholesterol (LDL-c) (41%), low high-density lipoprotein cholesterol (HDL-c) (45%), hypertriglyceridaemia (17%), dysglycaemia (9%), and metabolic syndrome (MS; 45%). The highest prevalence of unhealthy diet and PI was recorded in East Africa and Central Africa, respectively, whereas West Africa had the highest prevalence of high body mass index (BMI). Ethiopia had the highest prevalence of unhealthy diets, whereas Nigeria had the highest prevalence of stress and poor sleep. The healthcare sector had the highest cluster of risk factors and the highest prevalence of unhealthy diets. Only 5.7% of the studies implemented WWP, which had significant mitigating effects on most risk factors. This study concluded that the prevalence of most modifiable CVD risk factors is high among the corporate workforce in SSA, which is higher than that in the general population in most cases, and a well-designed WWP can significantly mitigate these risk factors.

## Introduction and background

Cardiovascular diseases (CVDs) have the highest mortality rate globally [1,2]. They account for approximately 33% (17.9 million) of global deaths annually [2,3]. However, all regions of the world are presently experiencing either a plateau or decline in the age-standardised CVD death rate, except for sub-Saharan Africa (SSA), where it is still increasing [4,5]. In 2013, CVD resulted in the death of 1 million sub-Saharan Africans, which is approximately 5.5% of the total global CVD deaths and 11.3% of all-cause mortality in Africa that year [5]. In addition, in 1990, the CVD death rate in SSA was only 1.2 times higher than that of all high-income regions combined, which increased to 2.1 times higher by 2019 [3]. Furthermore, CVD is currently the leading cause of death in people aged 45 years and above in Africa [6], and the CVD burden in Africa has been projected to at least double by 2030 [5].

Thus, SSA is presently experiencing an epidemic of CVD [5], which contributes to an increasing double disease burden and disease disparity in the region [7,8]. If unaddressed, this situation might significantly compromise the socio-economic growth of the region [9]. Different root factors have been implicated in the rising prevalence and burden of CVD in SSA, including changing age profiles and lifestyles in the region [2]. The increasing phenomena of Westernisation, urbanisation, and socio-economic development in SSA have changed the typical agrarian lifestyle and way of life of many people in the region to a more Western lifestyle [5,6]. The way people do business in SSA has also changed significantly, as evidenced by the increasing proliferation of industries and corporate companies, and the preference for white-collar jobs.

The corporate workplace environment has recently been classified as obesogenic because it often predisposes workers to obesity and other CVD risk factors [10,11]. Thus, assessing and mitigating modifiable CVD risk factors in the workplace has been identified as a vital strategy for reducing the burden of CVD globally [12,13]. However, while many studies have been conducted on the prevalence of CVD risk factors in SSA, most of them are hospital-based. Moreover, few systematic reviews are available on the efficacy of workplace wellness programmes (WWP) in mitigating CVD risk factors among corporate workers in SSA. This study investigated the prevalence and distribution of modifiable CVD risk factors among the corporate workforce in SSA in different regions, countries, and work sectors. This study also assessed the effects of WWP on CVD risk factors among the corporate workforce in SSA.

The study was registered with PROSPERO under the registration number CRD42024529666.

## Review

Methodology

The study was conducted in strict compliance with the Preferred Reporting Items for Systematic Reviews and Meta-Analyses (PRISMA) checklist and the PICO (Population, Investigation/Intervention, Comparison and Outcomes) protocol. The Google Scholar, Cochrane Library, PubMed, MEDLINE, Scopus, Impress and Science Direct) were carefully searched for relevant data from studies published between January 2010 and March 2024. Some of the search terms included cardiovascular disease risk factors, unhealthy diet, physical inactivity (PI), sedentary lifestyle, stress, poor sleep, tobacco smoking, alcohol abuse, overweight, obesity, hypertension, dysglycaemia, diabetes, dyslipidaemia, metabolic syndrome (MS), corporate workers, employees, sub-Saharan Africa and WWPs.

**Figure 1:**
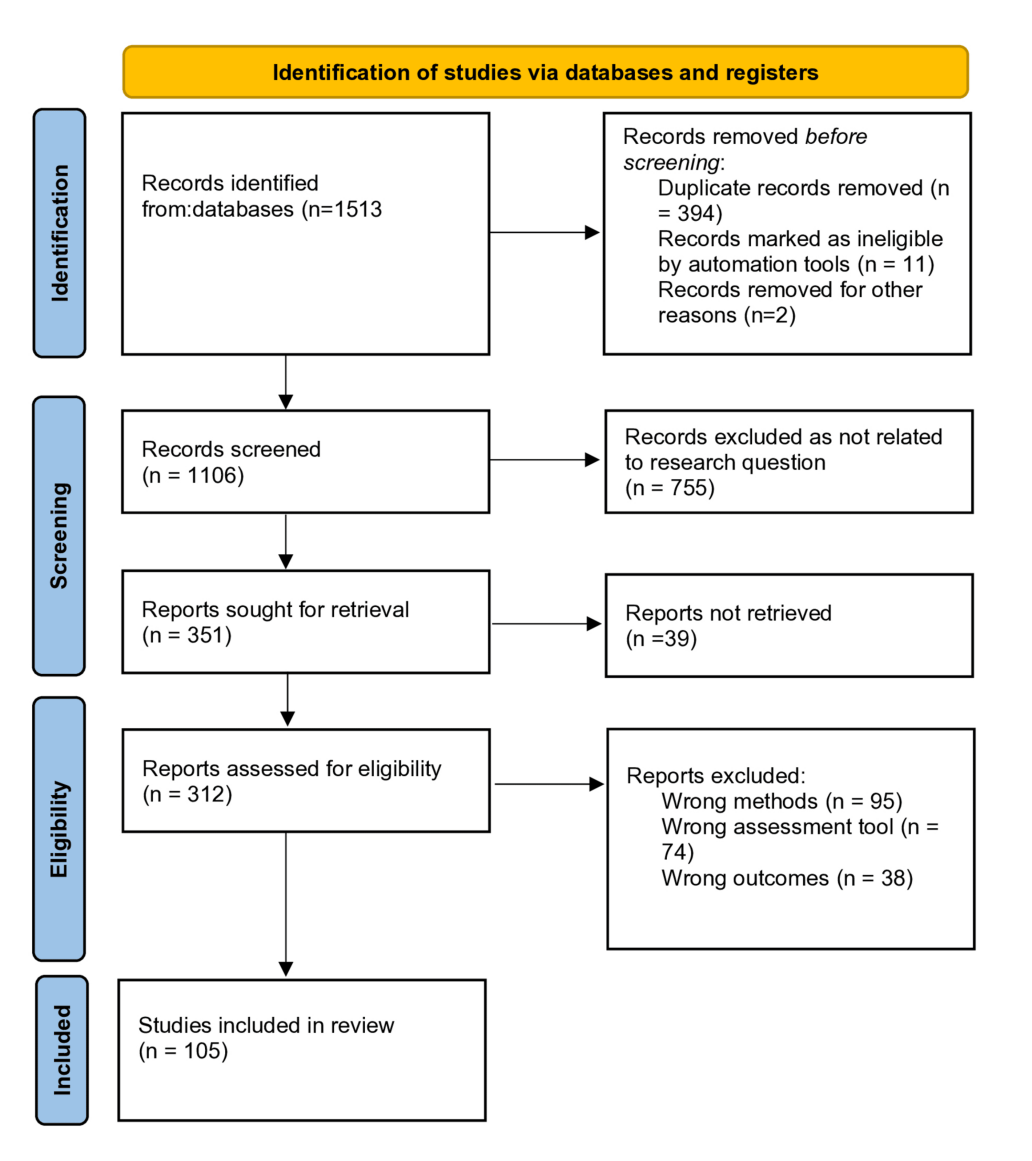
PRISMA flow diagram for the studies included in the systematic review and meta-analysis. PRISMA, Preferred Reporting Items for Systematic Reviews and Meta-Analyses

The search was performed by two independent reviewers between April and May 2024, and 1,513 related articles were identified. Subsequently, 394 duplicate articles were removed via the Rayyan App, 7,55 were removed for not meeting the inclusion criteria, and another 246 were screened out for not meeting the eligibility criteria. Consequently, the 105 most relevant articles were included in the systematic review and meta-analysis, as shown in the PRISMA chart (Figure 1).

Inclusion criteria

The inclusion criteria were as follows: participants working in a corporate business environment or undertaking a white-collar job; cross-sectional, case-control, and cohort studies, randomised controlled trials (RCTs), systematic reviews, and meta-analyses; studies on the prevalence of behavioural CVD risk factors (such as unhealthy diets, PI, stress, poor sleep, tobacco use, khat chewing, alcohol consumption, and harmful alcohol consumption) and intermediate CVD risk factors (including overweight, obesity, central obesity, high blood pressure, dysglycaemia, and MS); studies investigating the effects of WWPs on CVD risk factors; public or private corporate organisations (with more than 10 staff) with a physical location in any country from SSA; articles published between January 2010 and March 2024; and articles written in English.

Exclusion criteria

The exclusion criteria were as follows: patients managed in hospitals or other clinical settings; blue-collar workers (such as miners, construction workers, truck drivers, etc.); full-term athletes; case reports, case series, letters to the editor, commentaries, opinion papers, qualitative studies, conference proceedings, and policy papers; studies that focused solely on the levels of awareness, knowledge, perception, or attitudes towards CVD risk factors among participants; countries outside SSA; articles published before 2010; and publications in languages other than English.

Study selection and data extraction

All peer-reviewed published studies that investigated the prevalence of CVD risk factors and the effects of WWP on these risk factors among corporate workers in SSA were included. Case reports/series, letters to the editor, commentaries, opinion papers, qualitative studies, conference proceedings, policy papers and any studies that investigated only the levels of awareness, knowledge, perception and attitude toward CVD risk factors among participants were excluded.

Data collection process and data items

Two independent reviewers performed the database search and duplicate removal (via Rayyan App), and the extracted data were recorded in a standardised data abstraction form (Microsoft Excel®). The data collected included title, authors, year of publication, location (country, region and work sector) of the study, sample size, diagnostic criteria/assessment tools, nature of the intervention, outcomes and other key findings, and limitations of the studies. The Modified Newcastle-Ottawa scale was used to assess the quality of the included studies.

Study analysis

Review Manager (RevMan) 5.4 was used for data analysis. To calculate the overall prevalence of each risk factor, a meta-analysis was conducted considering studies that reported sample sizes and prevalence data across at least three studies. The summary statistics were then expressed as percentages with 95% confidence intervals (CIs). The risk-of-bias assessment was performed by using the Modified Newcastle-Ottawa scores scale (Table 1). Cochran's Q test assessed the statistical heterogeneity in effect sizes. A significant Q value suggests the presence of heterogeneity. Furthermore, the prevalence of total variance attributed to study heterogeneity was calculated using I2 statistics. The degree of heterogeneity was categorized as low, moderate, or high based on I2 values of <25%, 25%-75%, and above 75%, respectively. The statistical significance threshold was set at *P* < 0.05. Forest plots were utilized to represent the results visually.

**Table 1:** Modified Newcastle-Ottawa scores for the selected studies.

	Authors	Year	Modified Newcastle-Ottawa scores			
			Selection	Comparability	Outcome	Total
1	Onyango et al. (2017) [14]	2017	4	N/A	3	7
2	Geto et al. (2018) [15]	2018	3	N/A	3	6
3	Joshua and Kay (2014) [16]	2014	3		3	6
4	Ambakederemo and Chikezie (2018) [17]	2018	3	N/A	3	6
5	Richard et al. (2016) [18]	2016	4	N/A	3	7
6	Awosan et al. (2013) [19]	2013	4	N/A	3	7
7	Ajewole et al. (2017) [20]	2017	4	N/A	3	7
8	Hope (2023) [21]	2023	3	N/A	3	6
9	Dele-Ojo et al. (2021) [22]	2021	4	N/A	3	7
10	Shitu and Kassie(2021) [23]	2021	4	N/A	3	7
11	Haastrup et al. (2018) [24]	2018	4	N/A	3	7
12	James et al. (2018) [25]	2018	4	N/A	3	7
13	Travillet al. (2019) [26]	2019	3	N/A	3	6
14	Jingi et al. (2015) [27]	2015	4	N/A	3	7
15	Olaniyan et al. (2020) [28]	2020	4	N/A	3	7
16	Uwanuruochi et al. (2013) [29]	2013	3	N/A	3	6
17	Olaitanet al. (2020) [30]	2020	4	N/A	3	7
18	Adamu and Abdullahi (2017) [31]	2017	4	N/A	3	7
19	Gyang et al. (2018) [32]	2018	4	N/A	3	7
20	Alao et al. (2022) [33]	2022	4	N/A	3	7
21	Amougou et al. (2019) [34]	2019	4	N/A	3	7
22	Justice et al. (2021) [35]	2021	3	N/A	3	6
23	Odunaiya et al. (2020) [36]	2020	4	N/A	3	7
24	Bernard et al. (2023) [37]	2023	4	N/A	3	7
25	Khaild et al. (2022) [38]	2022	4	N/A	3	7
26	Adelowo and Sekoni (2013) [39]	2013	4	N/A	3	7
27	Akintunde et al. (2015) [40]	2015	3	N/A	3	6
28	Chukwuemeka et al. (2023) [41]	2023	4	N/A	3	7
29	Salaudeen et al. (2014) [42]	2014	3	N/A	3	6
30	Dele-Ojo et al. (2021) [43]	2021	3	N/A	3	6
31	Chinedu-Eleonu et al. (2021) [44]	2021	3	N/A	3	6
32	Buremoh et al. (2020) [45]	2020	4	N/A	3	7
33	Gebremariam et al. (2018) [46]	2018	3	N/A	3	6
34	Skaal and Pengpid (2011) [47]	2011	2	N/A	3	5
35	Iwuala et al. (2015) [48]	2015	4	N/A	3	7
36	Onowhakpor et al. (2018) [49]	2018	4	N/A	3	7
37	Ndejjo et al. (2015) [50]	2015	3	N/A	3	6
38	Atuahene et al. (2017) [51]	2017	4	N/A	3	7
39	Ajike and Ezeakunne (2020) [52]	2020	4	N/A	3	7
40	Muluvhu (2018) [53]	2018	4	N/A	3	7
41	Omosivie and Chibianotu (2020) [54]	2020	3	N/A	3	7
42	Sekoni et al. (2013) [55]	2013	4	N/A	3	7
43	Fadeyi et al. (2018) [56]	2018	3	N/A	3	6
44	Monakali et al. (2018) [57]	2018	3	N/A	3	6
45	Aladeniyi et al. (2017) [58]	2017	4	N/A	3	7
46	Hailu et al. (2023) [59]	2023	4	N/A	3	7
47	Diwe et al. (2015) [60]	2015	4	N/A	3	7
48	Adeolu et al. (2016) [61]	2016	4	N/A	3	7
49	Bappah et al. 2022) [62]	2022	4	N/A	3	7
50	Obiebi et al. (2020) [63]	2020	2	N/A	3	5
51	Yakubu and Bigelow (2019) [64]	2019	4	N/A	3	7
52	Mwagi (2018) [65]	2018	4	N/A	3	7
53	Obarisiagbon et al. (2018) [66]	2018	4	N/A	3	7
54	Badego et al. (2020) [67]	2020	4	N/A	3	7
55	Agyemang-Pambour et al. (2023) [68]	2023	4	N/A	3	7
56	Eze and Okorie (2024) [69]	2024	3	N/A	3	6
57	Okwor et al. (2020) [70]	2020	4	N/A	3	7
58	Adeyanju et al. (2023) [71]	2023	3	N/A	3	6
59	Oladimeji et al. (2014) [72]	2014	3	N/A	3	6
60	Paquissi et al. (2016) [73]	2016	2	N/A	3	5
61	Awunor and Isah (2015) [74]	2015	2	N/A	3	5
62	Alinaitwe et al. (2024) [75]	2024	4	N/A	3	7
63	Vincent-Onabajo et al. (2016) [76]	2016	3	N/A	3	6
64	Sumaila et al. (2016) [77]	2016	2	N/A	3	5
65	Akintunde et al. (2014) [78]	2014	3	N/A	3	6
66	Adaja and Idemudia (2018) [79]	2018	3	N/A	3	6
67	Addo et al. (2015) [80]	2015	4	N/A	3	7
68	Egbi et al. (2015) [81]	2015	4	N/A	3	7
69	Angaw et al. (2015) [82]	2015	3	N/A	3	6
70	Olagunju et al. (2021) [83]	2021	3	N/A	3	6
71	Oyeyemi and Adeyemi (2013) [84]	2013	3	N/A	3	6
72	Muluvhu et al. (2020) [85]	2020	3	N/A	3	6
73	Muluvhu et al. (2019) [86]	2019	3	N/A	3	6
74	Adebayo et al. (2020) [87]	2020	3	N/A	3	6
75	Hailu et al. (2022) [88]	2022	3	N/A	3	6
76	Segon et al. (2022) [89]	2022	4	N/A	3	7
77	Kolo et al. (2017) [90]	2017	4	N/A	3	7
78	Olubiyi et al. (2022) [91]	2022	3	0	3	6
79	Burger et al. (2016) [92]	2016	4	N/A	3	7
80	Jeanne and Stuart (2012) [93]	2012	3	N/A	3	6
81	Idris (2019) [94]	2019	3	N/A	3	6
82	Olawuyi and Adeoye (2018) [95]	2018	3	N/A	3	6
83	Enikuomehin et al. (2021) [96]	2021	3	N/A	3	6
84	Etim et al. (2018) [97]	2018	3	N/A	3	6
85	Owolabi et al. (2012) [98]	2012	2	N/A	3	5
86	Capingana et al. (2013) [99]	2013	4	N/A	3	7
87	Mekonen et al. (2020) [100]	2020	3	N/A	3	6
88	Aliyu et al. (2017) [101]	2017	4	N/A	3	7
89	Agyei et al. (2019) [102]	2019	3	N/A	3	6
90	Olatona et al. (2014) [103]	2014	4	N/A	3	7
91	Onyishi et al. (2022) [104]	2022	3	N/A	3	6
92	Chukwu (2023) [105]	2023	3	N/A	3	6
93	Makinde and Salawu (2021) [106]	2021	3	N/A	3	6
94	Ogba (2020) [107]	2020	3	N/A	3	6
95	Sime et al. (2022) [108]	2022	4	N/A	3	7
96	Bosu (2016) [109]	2016	4	N/A	3	7
97	Agbana et al. (2017) [110]	2017	3	2	3	8
98	Awosan et al. (2013) [111]	2013	3	2	3	8
99	Darcelle et al. (2020) [112]	2020	3	2	3	8
100	Abiodun (2021) [113]	2021	3	2	3	8
101	Adelowo et al. (2020) [114]	2020	3	2	3	8
102	Naila et al. (2013) [115]	2013	4	2	3	9
103	Torres et al. (2020) [116]	2020	3	2	3	8

To examine the potential presence of publication bias, the Egger test was employed, and a funnel plot was created to assess the distribution of study effects visually. Additionally, a leave-one-out sensitivity meta-analysis was conducted to evaluate the robustness of the findings and determine the influence of individual studies on the pooled estimates. This process involved systematically excluding each study from the analysis and recalculating the pooled estimates to evaluate their impact on the overall results. Moreover, the meta-analyses for prevalence were conducted using R version 4.3.3 (R Foundation for Statistical Computing) with the random effects model (REM) to address heterogeneity among studies.

Results

This study encompasses a total of 76,027 participants across 105 studies. Most of the studies (97, 92.4%) employed a cross-sectional design (14-108), while five (4.7%) were RCTs (112-116), and one (1%) each were systematic reviews (109), a nested case-control study (110), and a quasi-experimental study (111). The studies were conducted in Nigeria in 68 (64.8%), South Africa in 12 (11.4%), Ethiopia in 10 (9.5%), Ghana in 5 (4.8%), Cameroon in 2 (1.9%), Angola in 2 (1.9%), Kenya in 2 (1.9%), Uganda in 2 (1.9%) and Sudan in 1 (1%) (Table 2). Of the studies, only six (5.7%) conducted any form of intervention.

**Table 2:** Demographic characteristics and intervention across various studies.

Authors	Location (Region)	Sector of work	Study design	Sample size	Diagnostic criteria/assessment tools	Outcomes
Mwagi et al. (2017) [14]	Kenya (East Africa)	Telecommunication	Descriptive cross-sectional	370	Hypertension = ≥140/90 mmHg or history of hypertension	Hypertension = 30%
						Overweight = 49.5%
						Obesity = 25.1%
Zeleke (2018) [15]	Ethiopia (East Africa)	Healthcare	Descriptive cross-sectional	450	Hypertension ≥140/90 mmHg or history of hypertension	Current tobacco smoking = 4.2%
						Current alcohol drinking = 66.7%
						Physical inactivity = 4.8%
						Unhealthy diets = 99.6%
						Overweight = 37.1%
						Obesity = 6.9%
					Unhealthy diet = < 5 servings of fruits and vegetables/day	Central obesity = 80.2%
						Hypertension = 23.6%
					Total cholesterol (TC) ≥ 200 mg/dL	High TC = 28.2%
						High LDL = 25.1%
					LDL-cholesterol (LDL-c) >130 mg/dL	High TG = 19.3%
						Low HDL-c = 41.3%
					Triglycerides (TG) ≥ 150 mg/dL	Dyslipidaemia = 49.6%
						Dysglycaemia = 2.4%
Joshua and Kay (2014) [16]	Nigeria (West Africa)	Education	Descriptive cross-sectional	600	Exercise Stage Assessment Scale	Physical inactivity = 59.6%
Ambakederemo and Chikezie (2018) [17]	Nigeria (West Africa)	Healthcare	Descriptive cross-sectional	169	WHO STEPwise approach guidelines	Overweight = 39.1%
						Obesity = 13.6%
						Central obesity = 37.3%
						Hypertension = 22.1%
						Current smokers = 5.3%
						Current alcohol intake = 36.1%
						Physical inactivity = 74.6%
						No fruit intake/day = 74.6%
						Extra salt intake = 8.9%
						Unhealthy diet = 79.2%
Richard D, et al., 2016 [18]	Nigeria (West Africa)	Agro-Allied	Descriptive cross-sectional	510	WHO STEPwise approach guidelines.	Current Tobacco smokers = 6.9%
					Hypertension = ≥ 140/90 mmHg.	Harmful alcohol intake = 15.1%
						Unhealthy diet = 89.8%
						Physical inactivity = 66.5%
						Overweight = 13.9%
						Obesity = 3.9%
						Hypertension = 37.1%
Awosan et al. (2013) [19]	Nigeria (West Africa)	Education	Descriptive cross-sectional	110	WHO STEPwise approach guidelines	Unhealthy diet = 61.9%
						Physical inactivity = 5.7%
						Current Alcohol intake = 3.8%
						Current Tobacco smokers = 4.80%
						Stress = 52.4%
						Overweight = 22.9%
						Obesity = 26.7%
						Hypertension = 33.4%
						Diabetes = 10.5%
						High TC = 37.1%
Awosan et al. (2013) [19]	Nigeria (West Africa)	Finance	Descriptive cross-sectional	110	WHO STEPwise approach guidelines	Physical inactivity = 33.3%
						Overweight = 45.7%
						Obesity = 19.0%
						Current Tobacco smokers = 7.6%
						Hypertension = 22.9%
						Diabetes = 8.60%
						Unhealthy diet = 77.1%
						Alcohol intake = 27.6%
						High TC = 41.9%
						Stress = 43.8%
Ajewole et al. (2017) [20]	Nigeria (West Africa)	Education	Descriptive cross-sectional	203	Hypertension ≥ 140/90 mmHg	Hypertension = 5.8%
						Alcohol intake = 40.9%
						Unhealthy diet = 7.9%
						Current tobacco smokers = 9.4%
Hope (2023) [21]	Nigeria (West Africa)	Healthcare	Descriptive cross-sectional	140	Hypertension = JNC VII criteria	Hypertension = 32.1%
						Overweight = 53.6%
						Obesity = 23.6%
						Diabetes = 17.9%
						Alcohol intake = 27.9%
						Current tobacco smokers = 9.3%
						Physical inactivity = 80.0%
						Unhealthy diet = 20.0%
Bolade et al. (2021) [22]	Nigeria (West Africa)	Healthcare	Descriptive cross-sectional	248	Hypertension ≥140/90 mmHg	Obesity = 28.6%
						Central obesity = 60.9%
						Hypertension = 38.7%
						Dysglycaemia = 35.1%
						High TC = 50.4%
						High LDL-c = 37.9%
						Low HDL-c = 14.5%
						High TG = 24.2%
Kegnie and Ayenew (2021) [23]	Ethiopia (East Africa)	Finance	Descriptive cross-sectional	368	WHO STEPwise approach guidelines	Hypertension = 52.4%
						Overweight = 28.0%
						Obesity = 15.0%
						No vegetables/day = 15.2%
						No fruit/day = 6.5%
					General Physical Activity Questionnaire (GPAQ)	High salt intake = 43.5%
						Current alcohol intake = 76.1%
						Current Tobacco smokers = 4.4%
						Physical inactivity = 50.3%
						Stress = 65.2%
Haastrup et al. (2018) [24]	Nigeria (West Africa)	Administration	Descriptive cross-sectional	184	WHO STEPwise approach guidelines	Unhealthy diet = 90.8%
						Extra salt intake = 42.9%
						Current Tobacco smokers = 23.4%
						Passive Tobacco smokers = 18.4%
						Alcohol intake = 43.5%
						Harmful alcohol intake = 90.0%
						Physical inactivity = 64.1%
						Overweight = 42.4%
						Obesity = 14.7%
						Central obesity = 48.4%
						Hypertension = 33.7%
						Dysglycaemia = 24.0%
						High TC = 28.8%
James et al. (2018) [25]	Ghana (West Africa)	Healthcare	Descriptive cross-sectional	112	Hypertension = JNC VII criteria	Hypertension = 16.1%
						Overweight = 38.39%
						Obesity = 12.50%
						Dysglycaemia = 9.92%
						Dyslipidaemia = 26.79%
						High TC = 18.75%
						High TG = 10.71%
Andre et al. (2019) [26]	South Africa (Southern Africa)	Manufacturing	Descriptive cross-sectional	75	American College of Sports Medicine’s guidelines	Overweight = 32%
						Obesity = 32%
						Hypertension = 55%
						Dysglycaemia = 25.3%
						High TC = 28%
Ahmadou et al. (2015) [27]	Cameroon (Central Africa)	Healthcare	Descriptive cross-sectional	65	Hypertension = JNC VII criteria	Hypertension = 26.2%
					WHO STEPwise approach guidelines	Diabetes = 3.1%
						Overweight = 46.2%
						Obesity = 23.1%
						Current tobacco smokers = 12.3%
						Harmful alcohol intake = 61.5%
						Physical inactivity = 16.9%
Olaniyan et al. (2020) [28]	Nigeria (West Africa)	Mixed	Descriptive cross-sectional	260	Hypertension = JNC VII criteria	Hypertension = 40.4%
					WHO STEPwise approach guidelines	Obesity = 52.3%
						Central obesity = 35.0%
						Current tobacco smokers = 5.8%
						Alcohol intake = 26.5%
						Physical inactivity = 75.8%
						Diabetes = 38.1%
						Lipid profile
						High TC = 55.4%
						High LDL-c = 85.0%
						High TG = 3.1%
Uwanuruochi et al. (2013) [29]	Nigeria (West Africa)	Healthcare	Descriptive cross-sectional	299	Hypertension = WHO/ISH criteria	Hypertension = 37.5%
						Obesity = 42.1%
						Dysglycaemia = 20.8%
						High TC = 18.1%
					WHO STEPwise approach guidelines	High LDL-c = 26.8%
						Low HDL-c = 41.9%
						High TG = 9.7%
						Metabolic syndrome = 24.7%
Oluwasiji (2020) [30]	Nigeria (West Africa)	Healthcare	Descriptive cross-sectional	283	JNC VII criteria	Alcohol intake = 21.6%
						Current tobacco smoking = 2.5%
						Stress = 60.9%
						Physical inactivity = 55.1%
					International Stress Management Association Questionnaire (ISMAQ)	Diabetes = 1.4%
						Central obesity = 47.3%
						High BMI = 49.5%
						Hypertension = 30.1%
Adamu et al. (2017) [31]	Nigeria (West Africa)	Healthcare	Descriptive cross-sectional	196	Perceived stress screening tool	Stress = 55.9%
Gyang et al. (2018) [32]	Nigeria (West Africa)	Healthcare	Descriptive cross-sectional	155	Hypertension = >140/>90 mmHg	Hypertension = 41.9%
						Physical inactivity = 92.4%
					Poor sleep < 8 Hours/day	High BMI = 49.6%
						Central obesity = 58.8%
						Dysglycaemia = 45.8%
						Poor sleep = 48.9%
Alao et al. (2022) [33]	Nigeria (West Africa)	Healthcare	Descriptive cross-sectional	232	Stress = Work-Related Quality of Life (WRQoL) scale	Stress = 62.1%
Amougou et al. (2019) [34]	Cameroon (Central Africa)	Healthcare	Descriptive cross-sectional	350	WHO STEPwise approach guidelines	Obesity = 30.3%
						Central obesity = 46.9%
						Hypertension = 6.3%
					Unhealthy diet = <400 g of fruits and vegetables/day or <5 portions of fruits and vegetables/day	Diabetes = 2.9%
						Current tobacco smokers = 2.0%
						Unhealthy diet = 99.1%
Justice et al. (2021) [35]	Ghana (West Africa)	Finance	Descriptive cross-sectional	136	BMI: Weight/(Height)^2^	Overweight = 31.6%
						Obesity = 30.9%
Odunaiya et al. (2020) [36]	Nigeria (West Africa)	Healthcare	Descriptive cross-sectional	316	International Physical Activity Questionnaire (IPAQ)	Hypertension = 6.0%
						Overweight = 34.1%
						Obesity = 42.6%
						Physical inactivity = 49.1%
						Alcohol intake = 0.95%
Bernard Ubom et al. (2023) [37]	Nigeria (West Africa)	Healthcare	Descriptive cross-sectional	629	Validated self-developed tool using WHO guidelines	Physical inactivity = 73.1%
						Diabetes = 0.8%
						Current smoking = 3.9%
						Alcohol intake = 22.5%
Khaild et al. (2022) [38]	Sudan (East Africa)	Finance	Descriptive cross-sectional	98	High salt = Extra salt to meals	Hypertension = 45.9%
					Unhealthy diet = Fruits intake < 3 times/week	High BMI = 40.8%
						Current tobacco smokers = 20%
						High salt intake = 53.1%
						Unhealthy diet = 50%
						Physical inactivity = 38.8%
						Stress = 96%
Adelowo and Sekoni (2013) [39]	Nigeria (West Africa)	Finance	Descriptive cross-sectional	260	WHO STEPwise guidelines	Unhealthy diet = 7.3%
						High salt intake = 19.3%
						Current tobacco smokers = 10%
					Unhealthy diet = < 5 servings of fruits and/or vegetables/day	Alcohol intake = 42.7%
						Harmful alcohol intake = 27.1%
						Physical inactivity = 44.2%
Akintunde et al. (2015) [40]	Nigeria (West Africa)	Education	Descriptive cross-sectional	206	Heart Disease Fact Questionnaire (HDFQ)	Obesity = 38.3%
Chukwuemeka et al. (2023) [41]	Nigeria (West Africa)	Education	Descriptive cross-sectional	70	Cardiovascular Disease Risk Factors Knowledge Level (CARRF-KL) questionnaire	Hypertension = 12.9%
						Stress = 48.6%
						Overweight = 51.4%
					Cardiovascular risk factor (CRF) questionnaire	Obesity = 11.4%
						Central obesity = 21.4%
					ISMAQ	Physical inactivity = 18.6%
					IPAQ-SF	Dysglycaemia = 7.1%
						Current smokers = 7.1%
						Alcohol intake = 20.0%
Salaudeen et al. (2014) [42]	Nigeria (West Africa)	Finance	Descriptive cross-sectional	180	BMI = Weight/(Height)^2^	Current tobacco smokers = 32.2%
						Alcohol intake = 41.7%
						Unhealthy diet = 77.8%
						Physical inactivity = 75.0%
						Overweight = 14.4%
						Obesity = 20.0%
Dele-Ojo et al. (2021) [43]	Nigeria (West Africa)	Education	Descriptive cross-sectional	223	Heart Disease Fact Questionnaire (HDFQ)	Hypertension = 35.4%
					Hypertension = JNC VII criteria	Diabetes = 12.1%
						Overweight = 31.8%
						Obesity = 23.3%
					Unhealthy diet = < 5 servings of fruits/vegetables daily.	Physical inactivity = 83%
						Unhealthy diet = 67.7%
						Current tobacco smokers = 2.2%
Chinedu-Eleonu et al. (2021) [44]	Nigeria (West Africa)	Healthcare	Descriptive correlational	388	Hypertension ≥140/90 mmHg	Hypertension = 36.1%
Buremoh et al. (2020) [45]	Nigeria (West Africa)	Healthcare	Descriptive cross-sectional	196	Unhealthy diet = < 5 servings of fruits/vegetables/day	Unhealthy diet = 91.8%
						Physical inactivity = 77%
						Current smokers = 20.9%
						Alcohol intake = 21.9%
					Poor sleep < 5-6 hours/night	Poor sleep = 41%
						Central obesity = 58.2%
						Hypertension = 40.8%
						Dysglycaemia = 1.5%
Lemlem et al. (2018) [46]	Ethiopia (East Africa)	Mixed	Descriptive cross-sectional	1380	WHO STEPwise approach guidelines	Unhealthy diet = 99.7%
						Current smoker = 2.2%
						Khat chewing = 1.6%
						Alcohol intake = 18.9%
					Unhealthy diet = < 5 servings of fruits/vegetables daily	Physical inactivity = 41.0%
						Overweight = 26.0%
						Obesity = 4.1%
						Central obesity = 27.2%
					Physical inactivity = < 600 MET-minutes/week	High-systolic BP = 10.5%
						High-diastolic BP = 14.7%
						Dysglycaemia = 19.4%
						Diabetes = 40.9%
						High TC = 25.2%
						High LDL-c = 51.6%
						Low HDL-c = 59.2%
						High TG = 55.7%
Skaal and Pengpid (2011) [47]	South Africa (Southern Africa)	Healthcare	Descriptive cross-sectional	200	BMI = Weight/(Height)^2^	Diabetes = 10.0%
						Hypertension = 20.0%
						Stress = 32.5%
						Overweight = 26.5%
						Obesity = 47.0%
Iwuala et al. (2015) [48]	Nigeria (West Africa)	Healthcare	Descriptive cross-sectional	300	IPAQ-SF	Overweight = 44.7%
						Obesity = 27.3%
						Central obesity = 49.7%
						Physical inactivity = 79.2%
Onowhakpor et al. (2018) [49]	Nigeria (West Africa)	Healthcare	Descriptive cross-sectional	229	General Health Questionnaire (GHQ-12)	Stress = 50.7%
Ndejjo et al. (2015) [50]	Uganda (East Africa)	Healthcare	Descriptive cross-sectional, multicentre	200	National Institute of Occupational Safety and Health tool	Alcohol intake = 19.0%
						Physical inactivity = 59.0%
					Poor sleep <8 hours/day	Poor sleep = 75.0%
						Stress = 21.5%
Atuahene et al. (2017) [51]	Ghana (West Africa)	Mixed	Descriptive cross-sectional	271	BMI = Weight/(Height)^2^	Alcohol intake = 47.9%
						Current tobacco smoker = 2.7%
						Physical inactivity = 63.6%
						Overweight = 29.9%
						Obesity = 4.8%
Ajike et al. (2020) [52]	Nigeria (West Africa)	Finance	Descriptive cross-sectional	198	Validated self-developed structured tool	Stress = 94.4%
Muluvhu (2018) [53]	South Africa (Southern Africa)	Administration	Descriptive cross-sectional	535	IPAQ	Dysglycaemia = 25%
						Physical inactivity = 77%
						Overweight = 27%
					NCEP-ATPIII criteria	Obesity = 34%
						Central obesity = 64%
						Hypertension = 25%
					IDF diagnostic criteria.	Metabolic syndrome = 55%
						Alcohol intake = 29%
						Current tobacco smoker = 51%
Omosivie andChibianotu (2020) [54]	Nigeria (West Africa)	Judiciary	Descriptive cross-sectional	226	WHO STEPwise approach guidelines	Hypertension = 47.3%
						High BMI = 65.5%
					Unhealthy diet = Fruits/Vegetables intake < twice/week	Current smoker = 8.8%
						Alcohol intake = 28.8%
					High salt intake = Adding salt to food before eating or self-grading salt consumption as high	Unhealthy diet = 37.6%
						High salt intake = 30.5%
						Physical inactivity = 39.8%
						Diabetes = 10.2%
Sekoni et al. (2013) [55]	Nigeria (West Africa)	Finance	Descriptive cross-sectional	260	Hypertension ≥140/90 mmHg	Hypertension = 29.6%
					BMI: Weight/(Height)^2^	High BMI = 40.4%
Fadeyi et al. (2018) [56]	Nigeria (West Africa)	Healthcare	Descriptive cross-sectional	88	Poor sleep <7 hours/night	Current smoker = 1.1%
						Alcohol intake = 4.6%
						High BMI = 29.6%
						Poor sleep = 47.7%
Monakali et al. (2018) [57]	South Africa (Southern Africa)	Healthcare	Descriptive cross-sectional	203	Modified WHO STEPwise approach guidelines	Hypertension = 52%
						Alcohol intake = 26.6%
						Current smokers = 8.4%
						Physical inactivity = 32.5%
						Obesity = 46.8%
Aladeniyi et al. (2017) [58]	Nigeria (West Africa)	Mixed	Descriptive cross-sectional	4844	Modified WHO STEPwise approach guidelines	Hypertension = 35%
						Poor sleep = 85.2%
					Poor sleep <6 hours/day	Alcohol intake = 8.5%
						Physical inactivity = 60.6%
Hailu et al. (2023) [59]	Ethiopia (East Africa)	Manufacturing	Descriptive cross-sectional	370	Pittsburgh Sleep Quality Index (PSQI)	Overweight = 15.1%
					Food and Agriculture Organization’s Individual Dietary Diversity Score (IDDS)	Obesity = 4.1%
						Physical inactivity = 68.4%
						Current Tobacco smokers = 8.6%
						Khat chewing = 4.1%
						Alcohol intake = 52.4%
						Poor sleep = 75.4%
Diwe et al. (2015) [60]	Nigeria (West Africa)	Finance	Descriptive cross-sectional	194	Hypertension = <140/90mmHg.	Hypertension = 12.4%
						Alcohol intake = 50%
					BMI = Weight/(Height)^2^	Current Tobacco smokers = 8.3%
						Obesity = 37.5%
Adeolu et al. (2016) [61]	Nigeria (West Africa)	Healthcare	Descriptive cross-sectional	253	GHQ-12	Stress = 31.6%
					General practitioner job stress inventory	
Babangida et al. (2022) [62]	Nigeria (West Africa)	Education	Descriptive cross-sectional	281	Global Physical Activity Questionnaire (GPAQ)	Hypertension = 27.8%
						Overweight = 29.9%
						Obesity = 17.4%
						Current tobacco smokers = 12.1%
						Physical inactivity = 47.7%
Obiebi et al. (2020) [63]	Nigeria (West Africa)	Healthcare	Descriptive cross-sectional	232	HBP ≥140/90 mmHg	Hypertension = 36.2%
					BMI = Weight/(Height)^2^	Overweight = 41.4%
						Obesity = 20.3%
						Central obesity = 56.0%
						Physical inactivity = 26.3%
Yakubu and Bigelow (2019) [64]	Nigeria (West Africa)	Finance	Descriptive cross-sectional	3013	HBP ≥140/90 mmHg	Overweight = 39.3%
					BMI = Weight/(Height)^2^	Obesity = 23.6%
						Hypertension = 27.6%
Mwagi (2018) [65]	Kenya (East Africa)	Telecommunication	Descriptive cross-sectional	400	Modified WHO STEPwise approach guidelines	Hypertension = 29.7%
						Poor sleep = 22%
						Overweight = 40.3%
					Unhealthy diet = < 5 servings of fruits/vegetables daily	Obesity = 24.8%
						Unhealthy diet = 66.7%
						High salt intake = 60.4%
					Poor sleep ≤ 5 hours	Physical inactivity = 87.5%
						Alcohol intake = 55.0%
						Current tobacco smoker = 4.0%
Obarisiagbon et al. (2018) [66]	Nigeria (West Africa)	Agro-allied	Descriptive cross-sectional	354	WHO STEPwise approach guidelines	Hypertension = 18.4%
						Current tobacco smokers = 4.5%
						Alcohol intake = 48.6%
					Hypertension ≥140/90 mmHg	Overweight = 25.4%
						Obesity = 9.6%
						Diabetes = 2.8%
Badego et al. (2020) [67]	Ethiopia (East Africa)	Mixed	Descriptive cross-sectional	546	WHO STEPwise approach guidelines	Hypertension = 24.5%
					Unhealthy diet = Insufficient fruit intake ≤ 6 days/week and/or Insufficient vegetable intake ≤ 6 days/week	Diabetes = 5.7%
						Alcohol intake = 16.5%
						Current tobacco smokers = 0.4%
						Khat chewing = 12.6%
						Physical inactivity = 29.5%
						Insufficient fruits intake = 91.2%
						Insufficient vegetable intake = 67.3%
						High salt intake = 9.2%
						Overweight = 42.7%
						Obesity = 17.8%
Agyemang-Pambour et al. (2023) [68]	Ghana (West Africa)	Mixed	Descriptive cross-sectional	173	WHO STEPwise Approach guidelines	Hypertension = 29.3%
					GPAQ	Alcohol intake = 13.5%
					Hypertension = JNC VII criteria	Physical inactivity = 53.5%
						Overweight = 33.5%
						Obesity = 21.4%
Eze and Okorie (2024) [69]	Nigeria (West Africa)	Judiciary	Descriptive cross-sectional	120	Hypertension ≥140/90 mmHg	Hypertension = 25.8%
					Harmful alcohol Intake = intake of >14 units/ week in men and >7 units/ week in women	Overweight = 27.5%
						Obesity = 6.7%
						Dysglycaemia = 5.8%
						Dyslipidaemia = 3.3%
						Harmful alcohol intake = 5.0%
						Current tobacco smokers = 1.7%
Okwor et al. (2020) [70]	Nigeria (West Africa)	Finance	Descriptive cross-sectional	370	Stress = Health, Safety, Executive Management Standards Indicator Tool (HSE-MS IT)	Stress = 47%
Adeyanju et al. (2023) [71]	Nigeria (West Africa)	Mixed	Descriptive cross-sectional	296	Hypertension ≥140/90 mmHg	Hypertension = 33.4%
						Overweight = 43.6%
						Obesity = 15.2%
						Alcohol intake = 48.0%
						Current tobacco smokers = 7.4%
						Physical inactivity = 58.1%
Oladimeji et al. (2014) [72]	Nigeria (West Africa)	Mixed	Descriptive cross-sectional	801	Unhealthy diet = No fresh fruits and cooked vegetables/day	Hypertension = 29%
					PI < 30 minutes vigorous physical activity <5 days/week	Overweight = 35%
						Obesity = 27%
						Physical inactivity = 91%
						Unhealthy diet = 90%
						Current smokers = 6%
					Harmful alcohol intake ≥5 alcohol drinks intake at one sitting in males and ≥4 drinks in females	Harmful alcohol intake = 2%
Paquissi et al. (2016) [73]	Angola (Central Africa)	Education	Descriptive cross-sectional	781	Hypertension = JNC VII guideline	Hypertension = 17.9%
						Dysglycaemia = 10.6%
						Overweight = 34.4%
						Obesity = 19.9%
						Current smoking = 4.9%
						Harmful alcohol intake = 45.3%
Awunor and Isah (2015) [74]	Nigeria (West Africa)	Agro-Allied	Descriptive cross-sectional	349	Hypertension = WHO-ISH criteria	Hypertension = 28.1%
						Diabetes = 1.4%
						Obesity = 8.3%
						Alcohol intake = 49.0%
						Current smokers = 5.4%
						Physical inactivity = 43.8%
Alinaitwe et al. (2024) [75]	Uganda (East Africa)	Education	Descriptive cross-sectional	141	Modified WHO STEPwise approach guidelines	Hypertension = 26.2%
						Physical inactivity = 78.7%
						Overweight = 46.8%
						Obesity = 20.6%
						Unhealthy diet = 100%
						High salt intake = 46.8%
						Alcohol intake = 51.1%
Vincent-Onabajo et al. (2016) [76]	Nigeria (West Africa)	Education	Descriptive cross-sectional	441	Hypertension ≥140/90 mmHg	Hypertension = 36.1%
						Overweight = 39.9%
						Obesity = 22.2%
Sumaila et al. (2016) [77]	Nigeria (West Africa)	Healthcare	Descriptive cross-sectional	107	GPAQ	Hypertension = 26.2%
						Physical inactivity = 49.5%
						Unhealthy diet = 29.9%
						Current tobacco smokers = 12.2%
Akintunde et al. (2014) [78]	Nigeria (West Africa)	Education	Descriptive cross-sectional	206	Hypertension = JNC VII criteria	Hypertension = 40.8%
					Dyslipidaemia = NCEP panel IV guideline	Obesity = 38.3%
						Central obesity = 44.7%
						Dysglycaemia = 9.3%
						High TC = 49.5%
						High LDL-c = 48.1%
						Low HDL-c = 54.9%
Adaja and Idemudia 2018 [79]	Nigeria (West Africa)	Healthcare	Descriptive cross-sectional	325	Dyslipidaemia = NCEP panel guideline	Hypertension = 16.0%
					Unhealthy diet = No fruit and vegetable intake/day	Overweight = 31.7%
						Obesity = 25.5%
						Central obesity = 62.2%
						Alcohol intake = 53.5%
						Current tobacco smokers = 3.4%
						Physical inactivity = 68.3%
						Unhealthy diet = 68.9%
						Hight TC = 43.4%
						High LDL-c = 56.0%; low HDL-c = 82.2%
						High TG = 5.5%
Addo et al (2015) [80]	Ghana (West Africa)	Finance	Descriptive cross-sectional	180	WHO STEPwise approach guidelines	Overweight = 37.8%
						Obesity = 17.8%
					General Practice Physical Activity Questionnaire (GPPAQ).	Alcohol intake = 57.8%
						Physical inactivity = 83.3%
Egbi et al. (2015) [81]	Nigeria (West Africa)	Healthcare	Descriptive cross-sectional	231	Hypertension ≥140/90 mmHg	Hypertension = 21.3%
						Overweight = 35.5%
						Obesity = 23.8%
						Central obesity = 13.9%
						Alcohol intake = 23.8%
						Current smokers = 2.2%
						Dysglycaemia = 2.7%
Angaw et al. (2015) [82]	Ethiopia (East Africa)	Mixed	Descriptive cross-sectional	629	WHO STEPwise approach guidelines	Hypertension = 27.3%
					Unhealthy diet = Fruits and vegetable intake < 4 times/week	Current Tobacco smokers = 4.8%
						Khat chewing = 5.0%
						Insufficient fruits intake = 93.6%
						Insufficient vegetable intake = 80.2%
Olagunju et al. (2021) [83]	Nigeria (West Africa)	Healthcare	Descriptive cross-sectional	303	GHQ-12	Stress = 23.4%
					PSQI	Poor sleep = 60.4%
Oyeyemi and Adeyemi (2013) [84]	Nigeria (West Africa)	Mixed	Descriptive cross-sectional	292	IPAQ-SF	History of hypertension = 23.1%
						Metabolic syndrome = 48.8%
						Obesity = 24.0%
						Physical inactivity = 58.5%
Muluvhu et al. (2020) [85]	South Africa (Southern Africa)	Mixed	Descriptive cross-sectional	468	Physical Activity Index (PAI) questionnaire	Hypertension = 25%
						Overweight = 26.0%
						Obesity = 33%
						Alcohol intake = 29%
						Metabolic syndrome = 55%
						Current tobacco smokers = 51%
						Physical inactivity = 77%
Muluvhu et al. (2019) [86]	South Africa (Southern Africa)	Mixed	Descriptive cross-sectional	452	HBP ≥140/90 mmHg	Hypertension = 25%
					BMI = Weight/(Height)^2^	Overweight = 27%
						Obesity = 34%
Adebayo et al. (2020) [87]	Nigeria (West Africa)	Education	Descriptive cross-sectional	847	Hypertension ≥140/90 mmHg	Hypertension = 15.0%
						Overweight = 32%
						Obesity = 25.5%
						Physical inactivity = 86%
					Unhealthy diet = No daily fruit and vegetable intake/day.	Current tobacco smokers = 1.5%
						Alcohol intake = 24%
						No fruit intake/day = 75.5%
						No vegetable intake/day = 81%
Hailu et al. (2022) [88]	Ethiopia (East Africa)	Education	Descriptive cross-sectional	607	PSQI.	High BMI = 20.6%
					Workplace Stress Scale	Poor sleep = 60.3%
						Current tobacco smokers = 17.8%
						Khat chewing = 3.1%
						Alcohol intake = 18.5%
						Physical inactivity = 66.7%
						Stress = 45.5%
Segon et al. (2022) [89]	Ethiopia (East Africa)	Healthcare	Descriptive cross-sectional	510	PSQI	Poor sleep = 75.5%
					DASS-21	Stress = 33.1%
					ASSIST questionnaire	Current tobacco smokers = 8.4%
						Khat chewing = 9.6%
						Alcohol intake = 33.1%
Kolo et al. (2017) [90]	Nigeria (West Africa)	Healthcare	Descriptive cross-sectional	160	PSQI	Poor sleep = 54.2%
Olubiyi et al. (2022) [91]	Nigeria (West Africa)	Healthcare	Descriptive cross-sectional	301	WHO STEPwise approach	High TC = 62.8%
						High LDL-c = 26.6%
					Dyslipidaemia = NCEP panel guideline	Low HDL-c = 7.3%
						High TG = 10.3%
Burger et al. (2016) [92]	South Africa (Southern Africa)	Agro-Allied	Descriptive cross-sectional correlational	118	General Health Questionnaire (GHQ)	Alcohol intake = 34.7%
						Current tobacco smokers = 33.9%
Jeanne and Stuart (2012) [93]	South Africa (Southern Africa)	Administration (Hospitality)	Descriptive cross-sectional	137	Hypertension ≥140/90 mmHg	Hypertension = 34.3%
Idris (2019) [94]	Nigeria (West Africa)	Finance	Descriptive cross-sectional	52	HBP ≥140/90 mmHg	Hypertension = 28.8%
					BMI = Weight/(Height)^2^	Overweight = 50%
						Obesity = 15.4%
Olawuyi and Adeoye (2018) [95]	Nigeria (West Africa)	Administration	Descriptive cross-sectional	606	WHO STEPwise approach guidelines	Hypertension = 33.1%
					IPAQ.	High BMI = 57.3%
					Unhealthy diet < 5 servings of fruits and vegetables/day	Central obesity = 37.1%
					Harmful alcohol intake = > 5 drinks in men or >4 drinks in women on one or more occasions within 30 days	Current tobacco smokers = 6.5%
						Alcohol abuse = 7.8% Physical inactivity = 62.2%
						Unhealthy diet = 69.7%
						Dysglycaemia = 7.1%
Enikuomehin et al. (2021) [96]	Nigeria (West Africa)	Healthcare	Descriptive cross-sectional	192	Hypertension ≥140/90 mmHg	Hypertension = 22.4% Overweight = 26.0 %
					Dyslipidaemia = NCEP panel guideline	Obesity = 11.5%
					Unhealthy diet = No fruits and vegetables/day	Central obesity = 16.7%
					Physical inactivity = < 30 minutes/day	Physical inactivity = 24%
						Unhealthy diet = 73.4%
						Dysglycaemia = 3.1%
Etim et al. (2018) [97]	Nigeria (West Africa)	Healthcare	Descriptive cross-sectional	198	Stress assessment/workload analysis questionnaire	Stress = 92.8%
Owolabi et al. (2012) [98]	Nigeria (West Africa)	Healthcare	Descriptive cross-sectional	324	Hypertension ≥140/90 mmHg	Hypertension = 20.1%
					Job demand control questionnaire	Overweight = 24.7%
						Obesity = 9.9%
						Physical inactivity = 43.5%
						Alcohol intake = 6.5%
						Current Tobacco smokers = 24.4%
						Stress = 26.2%
Capingana et al. (2013) [99]	Angola (Central Africa)	Education	Descriptive cross-sectional	615	Modified WHO-MONICA Project questionnaire	Hypertension = 45.2%
						Overweight = 29.3%
					Modified WHO STEPwise guidelines	Obesity = 19.6%
						Current tobacco smokers = 7.2%
					High TC ≥ 240 mg/dL	Physical inactivity = 87.2%
						Diabetes = 5.7%
					High LDL-c ≥ 160 mg/dL	High TC = 11.1%
						High LDL-c = 19.8%
					High TG = ≥ 150 mg/dl	Low HDL-c = 50.1%
						High TG = 10.6%
Mekonen et al. (2020) [100]	Ethiopia (East Africa)	Finance	Descriptive cross-sectional	285	Workplace stress assessment scale	Overweight = 20.0%
						Obesity = 5.3%
						Alcohol intake = 45.6%
						Current tobacco smokers = 1.1%
						Physical inactivity = 66.3%
						Stress = 21.1%
Aliyu et al. (2017) [101]	Nigeria (West Africa)	Healthcare	Descriptive cross-sectional	100	PSQI	Poor sleep = 61%
Agyei et al. (2019) [102]	Nigeria (West Africa)	Education	Descriptive cross-sectional	153	Effort-Reward Imbalance (ERI) Scale	Stress = 58.82%
Agyei et al. (2019) [102]	South Africa (Southern Africa)	Education	Descriptive cross-sectional	153	Effort-Reward Imbalance (ERI) Scale	Stress = 59.18%
Olatona et al. (2014) [103]	Nigeria (West Africa)	Finance	Descriptive cross-sectional	223	ISMA Questionnaire	Stress = 91.5%
Onyishi et al. (2022) [104]	Nigeria (West Africa)	Mixed	Descriptive cross-sectional	3,572	Depression, Anxiety, and Stress Scale - 21 items (DASS-21)	Stress = 94.13%
Chukwu (2023) [105]	Nigeria (West Africa)	Healthcare	Descriptive cross-sectional	270	Self-structured validated questionnaire	Stress = 86.3%
Makinde and Salawu (2021) [106]	Nigeria (West Africa)	Healthcare	Descriptive cross-sectional	196	Expanded Nursing Stress Scale (ENjSS)	Stress: 82.1%
Ogba (2020) [107]	Nigeria (West Africa)	Healthcare	Descriptive cross-sectional	337	Occupational Stress Index scale	Stress = 64.5%
Sime et al. (2022) [108]	Ethiopia (East Africa)	Manufacturing (Textile)	Descriptive cross-sectional	413	Workplace Stress Scale (WPSS)	Stress = 47.5%
						Alcohol intake = 37.0%
						Khat chewing = 12.1%
Bosu (2016) [109]	West Africa (West Africa)	Mixed	Systematic review	34,919		Obesity
						Healthcare = 42.1%
						Telecom = 97.7%
Agbana et al. (2017) [110]	Nigeria (West Africa)	Agro-Allied	Nested case-control	510	WHO STEPwise guidelines	Stress = 44.1%
Awosan et al. (2013) [111]	Nigeria (West Africa)	Education	Quasi-experimental (Pre-test Post-test)	216	WHO STEPwise approach guidelines	Baseline
						Hypertension = 29.6%
						Diabetes = 10.2%
						High TC = 89.8%
						Physical inactivity = 21.3%
						Current tobacco smokers = 3.7%
						Unhealthy diet = 68.5%
						Overweight = 28.7%
						Obesity = 13.0%
						Mean SBP = 115.84 mmHg
						Mean DBP = 78.02 mmHg
						Mean weight = 68.65 kg
						Mean FBS = 85.13 mg/dL
						Mean TC = 185.23 mg/dL
						Follow-up
						Hypertension = 17.8%
						Diabetes = 3.0%
						High TC = 68.4%
						Physical inactivity = 6.9%
						Current tobacco smokers = 3.0%
						Unhealthy diet = 31.7%
						Overweight = 28.7%
						Obesity = 10.9%
						Mean SBP = 112.97 mmHg
						Mean DBP = 76.54 mmHg
						Mean weight = 67.64 kg
						Mean FBS = 78.44 mg/dL
						Mean TC = 172.52 mg/dL
Darcelle et al. (2020) [112]	South Africa (Southern Africa)	Administration (Energy)	Randomised controlled trial	137	GPAQ.	Baseline: Alcohol intake = 78.2%
					AUDIT-10.	Harmful alcohol intake = 21.8%
					Unhealthy diet = < 5 portions of fruits and vegetables/day.	Current smoking = 25.0%
					PI = < 600 MET minutes/week.	Unhealthy diet = 73.2%
					The 12-item stress screening tool	Physical inactivity = 55.9%
						Diabetes = 6.0%
						Hypertension = 15.9%
						High TC = 16.4%
						Stress level = 17.4%
						Mean SBP = 131.6mmHg
						Mean DBP = 83.4 mmHg
						Mean TC = 5.6 mmol/L
						Mean RBS = 5.7 mmol/L
						Mean BMI = 29.0
						Mean waist circumference = 92.1 cm
						Follow-up
						Alcohol intake = 93.5%
						Harmful alcohol intake = 6.4%
						Current tobacco smoking = 21.8%
						Unhealthy diet = 35.8%
						Physical inactivity = 34.7%
						Diabetes = 9.0%
						Hypertension = 18.9%
						High TC = 14.9%
						Stress level: 13.3%
						Mean SBP = 121.4 mmHg
						Mean DBP = 79.5 mmHg
						Mean TC = 5.1 mmol/L
						Mean RBS = 6.0 mmol/L
						Mean BMI = 29.0
						Mean waist circumference = 92.2 cm
Abiodun (2021) [113]	Nigeria (West Africa)	Administration	Randomised controlled trial	178	WHO STEPwise approach guidelines	Baseline
						Unhealthy diet = 92.0%
						High salt intake = 45.4%
						Current smokers = 21.6%
						Passive smokers =27%
						Physical inactivity = 68.2%
						Mean BMI = 26.98
						Mean waist circumference = 90.0 cm
						Mean SBP = 125.43 mmHg
						Mean DBP = 80.48 mmHg
						Mean FBS = 5.32 mmol/L
						Mean TC = 4.97 mmol/L
						Total CVD risk score = 5.45
						Follow-up
						Unhealthy diet = 22.7%
						High salt intake = 5.7%
						Current tobacco smokers = 10.2%
						Passive smokers = 3.2%
						Physical inactivity = 29.5%
						Mean BMI = 25.20
						Mean waist circumference = 89.1 cm
						Mean SBP = 119.91 mmHg
						Mean DBP = 76.07 mmHg
						Mean FBS = 4.96 mmol/L
						Mean TC =4.56 mmol/L
						Total CVD risk score = 5.11
Adelowo et al. (2020) [114]	Nigeria (West Africa)	Administration	Randomised controlled trial	88	Finnish Diabetes Risk Score (FINDRISC) questionnaire	Baseline
						Physical inactivity = 68.2%
						Unhealthy diet = 92%
						Mean BMI = 26.98
						Mean waist circumference = 90.0 cm
						Diabetes risk score = 7.82
						Follow-up
						Physical inactivity = 29.5%
						Unhealthy diet = 22.7%
						Mean BMI = 25.2
						Mean waist circumference = 89.1 cm
						Diabetes risk score = 6.06
Naila et al. (2013) [115]	South Africa (Southern Africa)	Manufacturing (Textile)	Randomised Controlled Trial	80	Health-Related Quality of Life (HRQoL EQ-5D) questionnaire	Increased strength/stretch exercise = 70%
						Increased walking exercise = 77%
						Increased swimming exercise = 60%
					Stanford Exercise Behaviours Scale	Increased cycling exercise = 100%
						Increased in other aerobic exercises = 81%
						Reduction in BMI = 89%
Torres et al. (2020) [116]	South Africa (Southern Africa)	Finance	Randomised Controlled Trial	251	ACSM FITT-VP criteria	Baseline
						Hypertension = 16.3%
						Obesity = 39.4%
						Physical inactivity = 55.4%
						Dyslipidaemia = 6.4%
						Dysglycaemia = 3.6%
						Current tobacco smokers = 3.6%
						Mean BMI = 28.2
						Mean waist circumference = 93.7 cm
						Mean SBP = 121.3 mmHg
						Mean DBP = 78.9 mmHg
						Follow-up
						Hypertension = 10.8%
						Obesity = 32.7%
						Physical inactivity = 2.0%
						Dyslipidaemia = 6.4%
						Dysglycaemia = 3.6%
						Current tobacco smokers = 2.0%
						Mean BMI = 26.6
						Mean waist circumference = 91.5 cm
						Mean SBP = 120.9 mmHg
						Mean DBP = 76.8 mmHg

Prevalence of behavioural CVD risk factors among the corporate workforce in SSA

**Figure 2:**
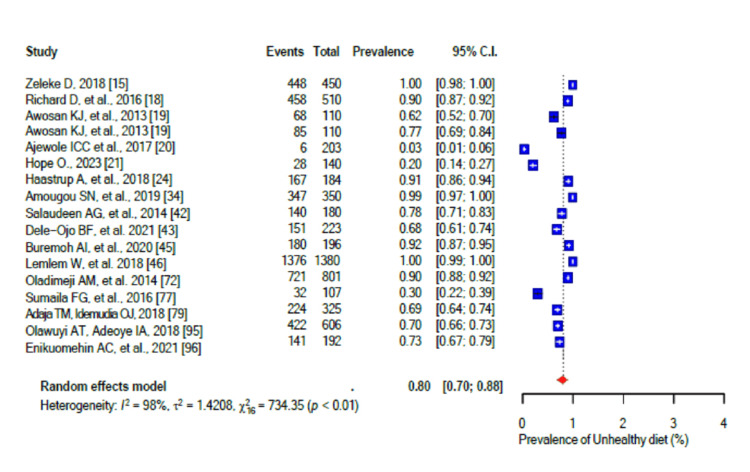
Forest plot of the prevalence of a generally unhealthy diet among the corporate workforce in Sub-Saharan Africa.

**Figure 3:**
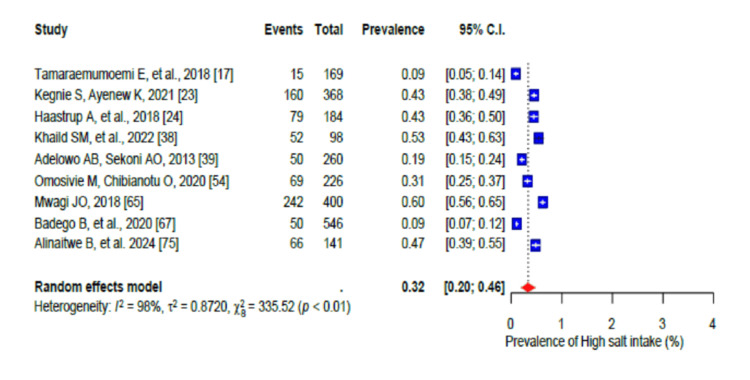
Forest plot of the prevalence of regular high salt intake among the corporate workforce in Sub-Saharan Africa.

**Figure 4:**
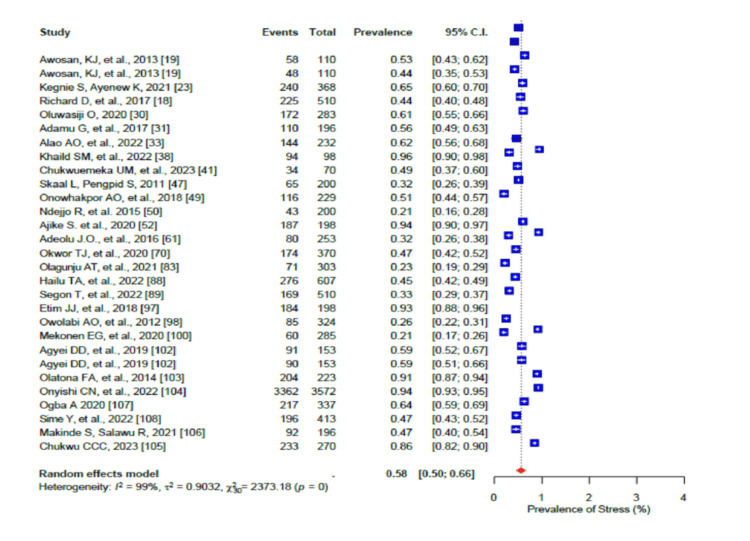
Forest plot of the prevalence of stress among the corporate workforce in Sub-Saharan Africa.

**Figure 5:**
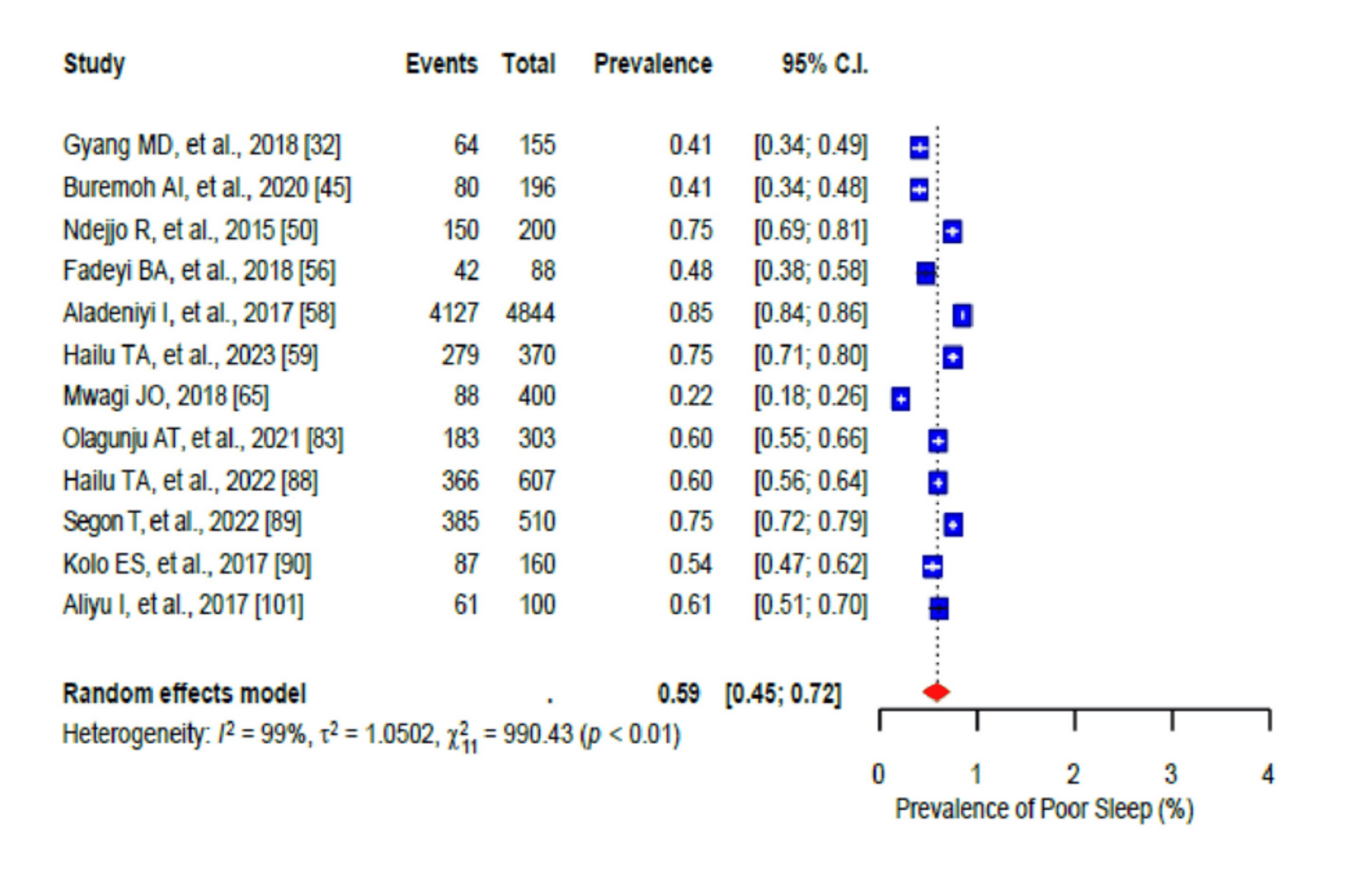
Forest plot of the prevalence of poor sleep among the corporate workforce in Sub-Saharan Africa.

The pooled prevalence of a generally unhealthy diet was 80% (95% CI: 0.70-0.88), with a high level of heterogeneity (I2 = 98%) (Figure 2). Furthermore, the high salt intake pooled prevalence was 32% (95% CI: 0.20-0.46; I2 = 98%) (Figure 3). In addition, stress was prevalent in 58% (95% CI: 0.50-0.66) of the population with a high level of heterogeneity (I2 = 96%-99%) (Figure 4), while the pooled prevalence of poor sleep was 59% (95% CI: 0.45-0.72), also with a high level of heterogeneity (I2 = 96-99%) (Figure 5).

**Figure 6:**
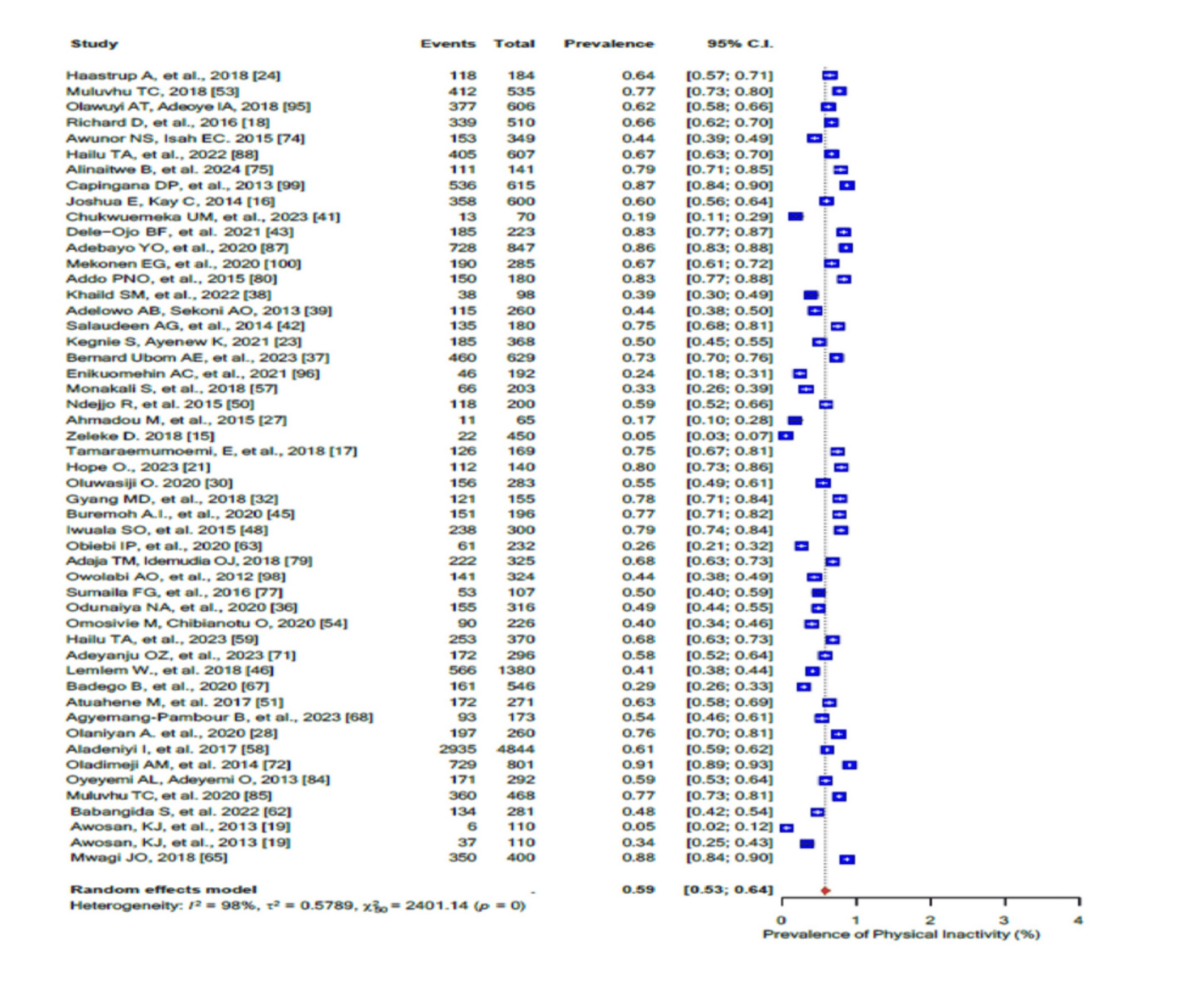
Forest plot of the prevalence of physical inactivity among the corporate workforce in Sub-Saharan Africa.

**Figure 7:**
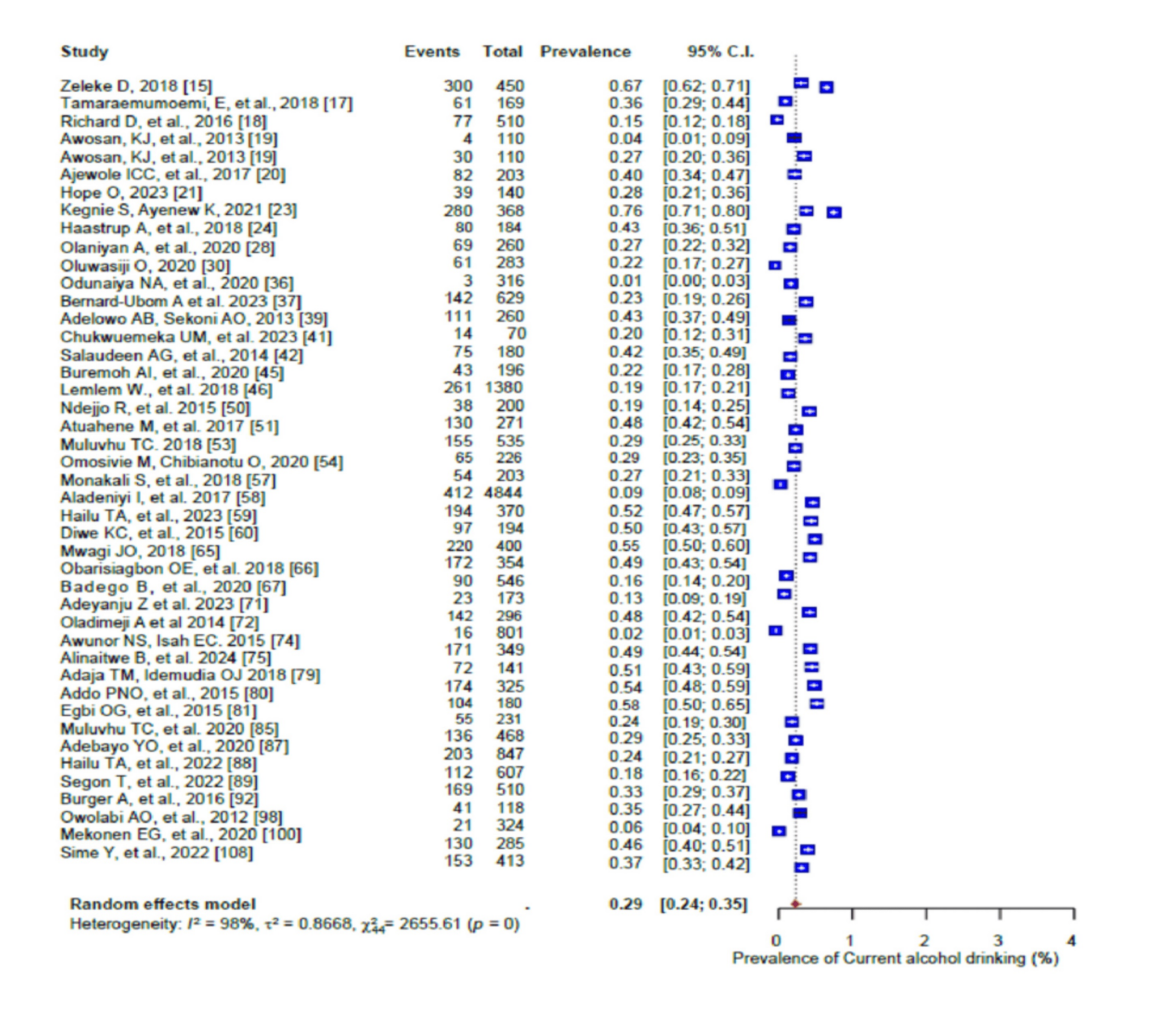
Forest plot of the prevalence of current alcohol consumption among the corporate workforce in Sub-Saharan Africa.

**Figure 8:**
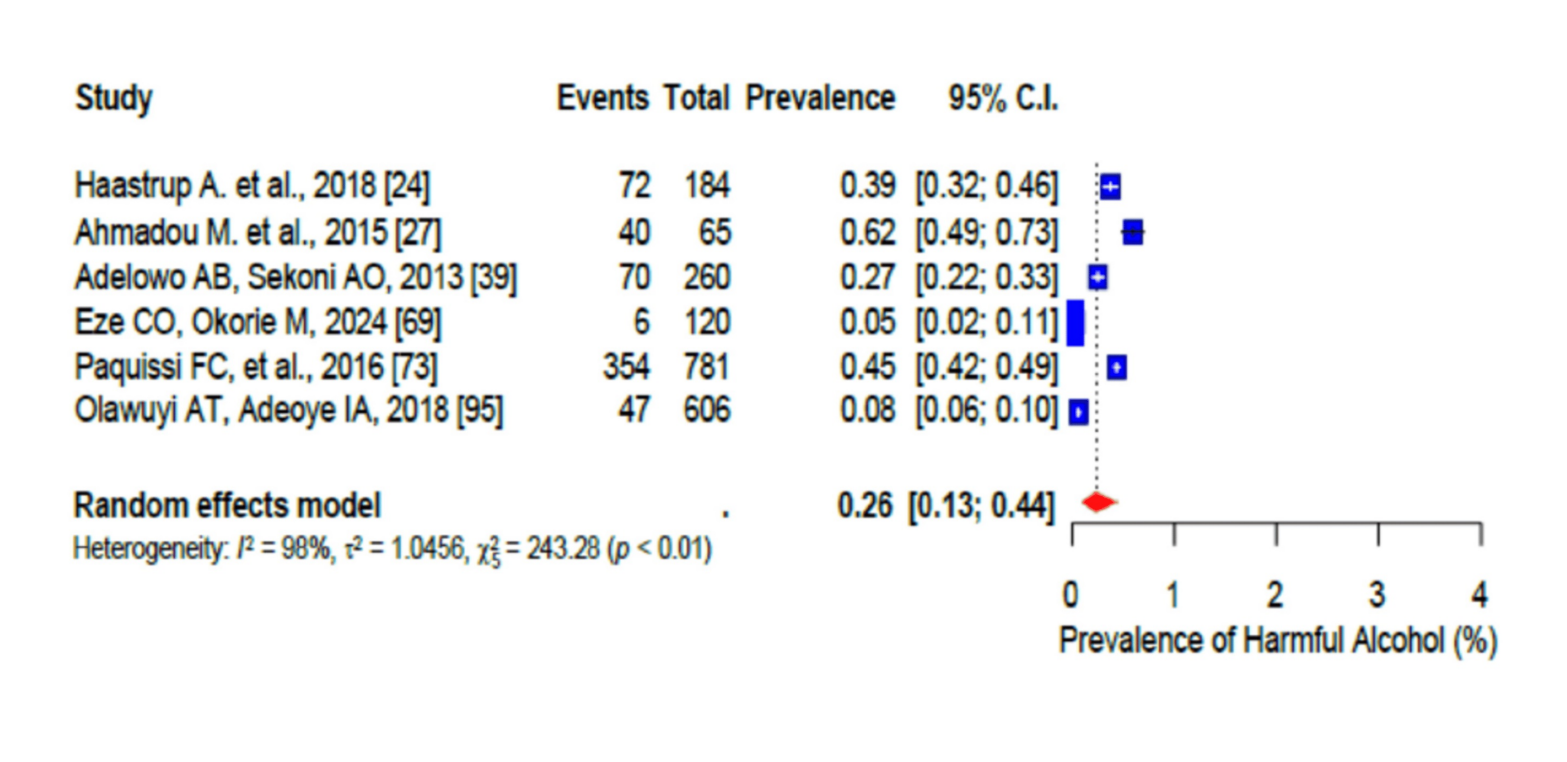
Forest plot of the prevalence of harmful alcohol consumption among the corporate workforce in Sub-Saharan Africa.

**Figure 9:**
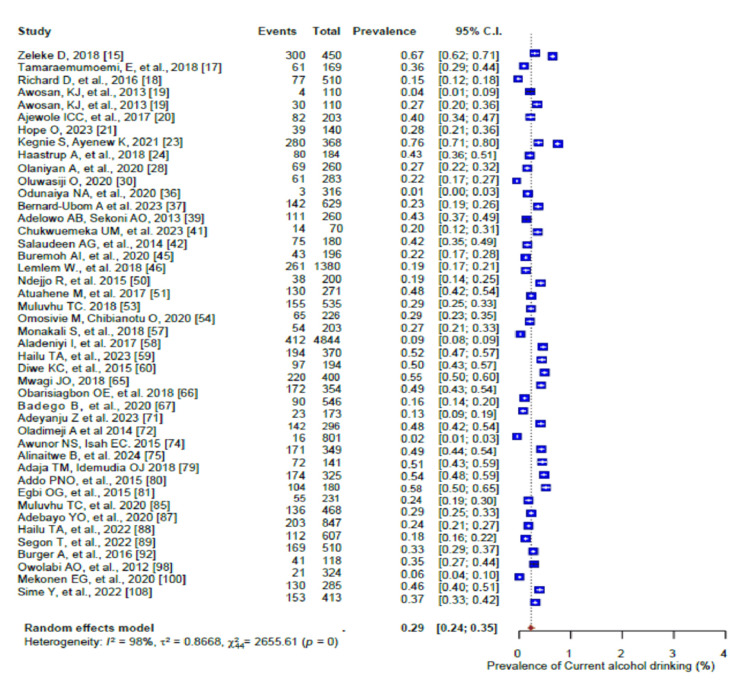
Forest plot of the prevalence of current tobacco smoking among the corporate workforce in Sub-Saharan Africa.

**Figure 10:**
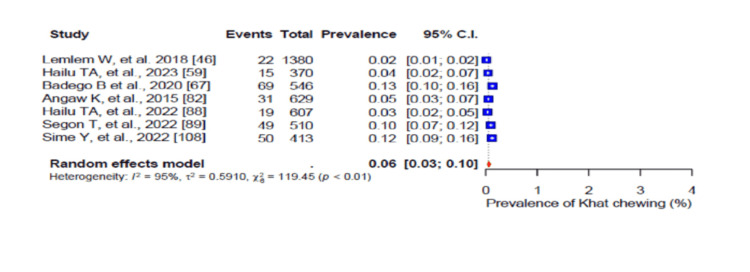
Forest plot of the prevalence of khat chewing among the corporate workforce in Sub-Saharan Africa.

In addition, 59% (95% CI: 0.53-0.64; I2 = 98%) of the participants were physically inactive (Figure 6). The pooled prevalence of current alcohol consumption was 29% (95% CI: 0.24-0.35) (I2 = 97%-98%) (Figure 7), while the pooled prevalence of harmful alcohol consumption was 26% (95% CI: 0.13-0.44) (I2 = 98%-99%) (Figure 8). However, the pooled prevalence of current tobacco use/smoking was only 7% (95% CI: 0.5-10.0) (Figure 9), while khat chewing was reported in only 6% of the participants (95% CI: 0.3-0.10; I2 = 95%) (Figure 10).

Prevalence of intermediate CVD risk factors among the corporate workforce in SSA

**Figure 11:**
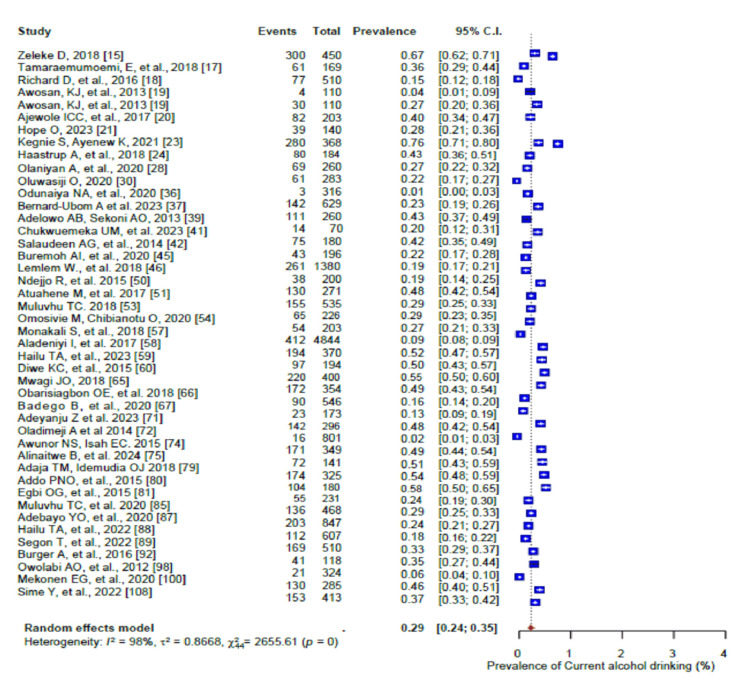
Forest plot of the prevalence of overweight among the corporate workforce in Sub-Saharan Africa.

**Figure 12:**
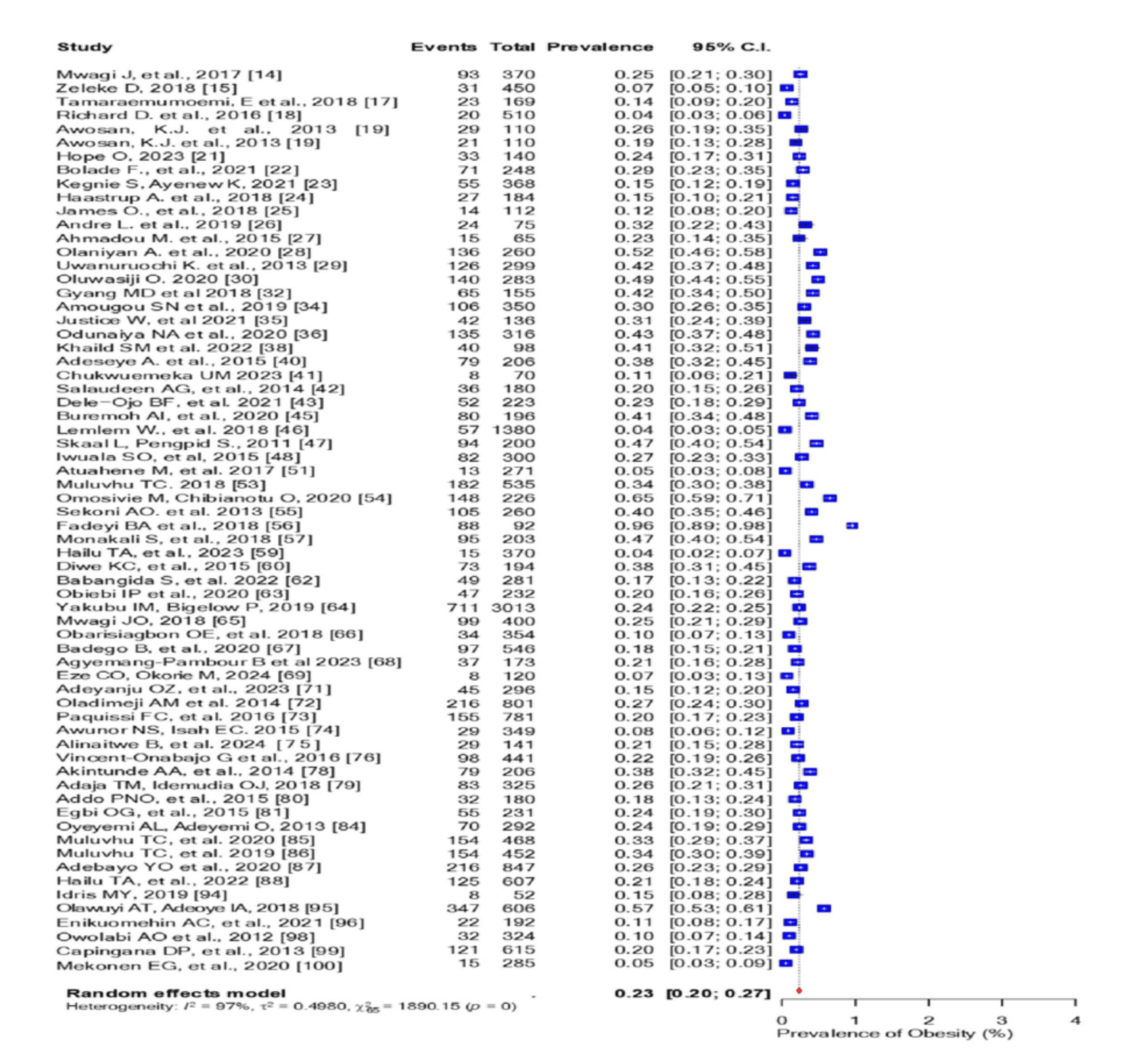
Forest plot of the prevalence of obesity among the corporate workforce in Sub-Saharan Africa.

**Figure 13:**
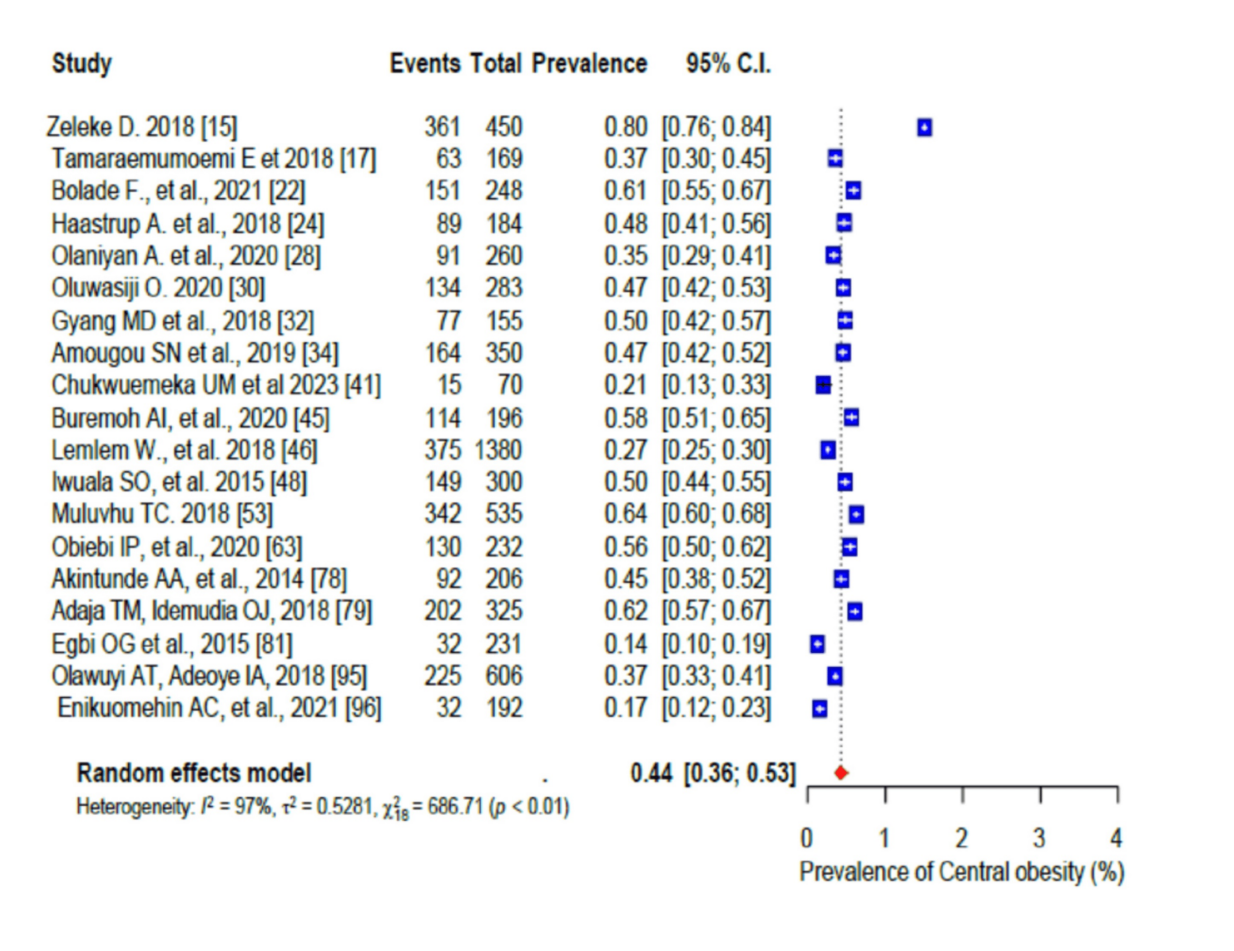
Forest plot of the prevalence of central obesity among the corporate workforce in Sub-Saharan Africa.

**Figure 14:**
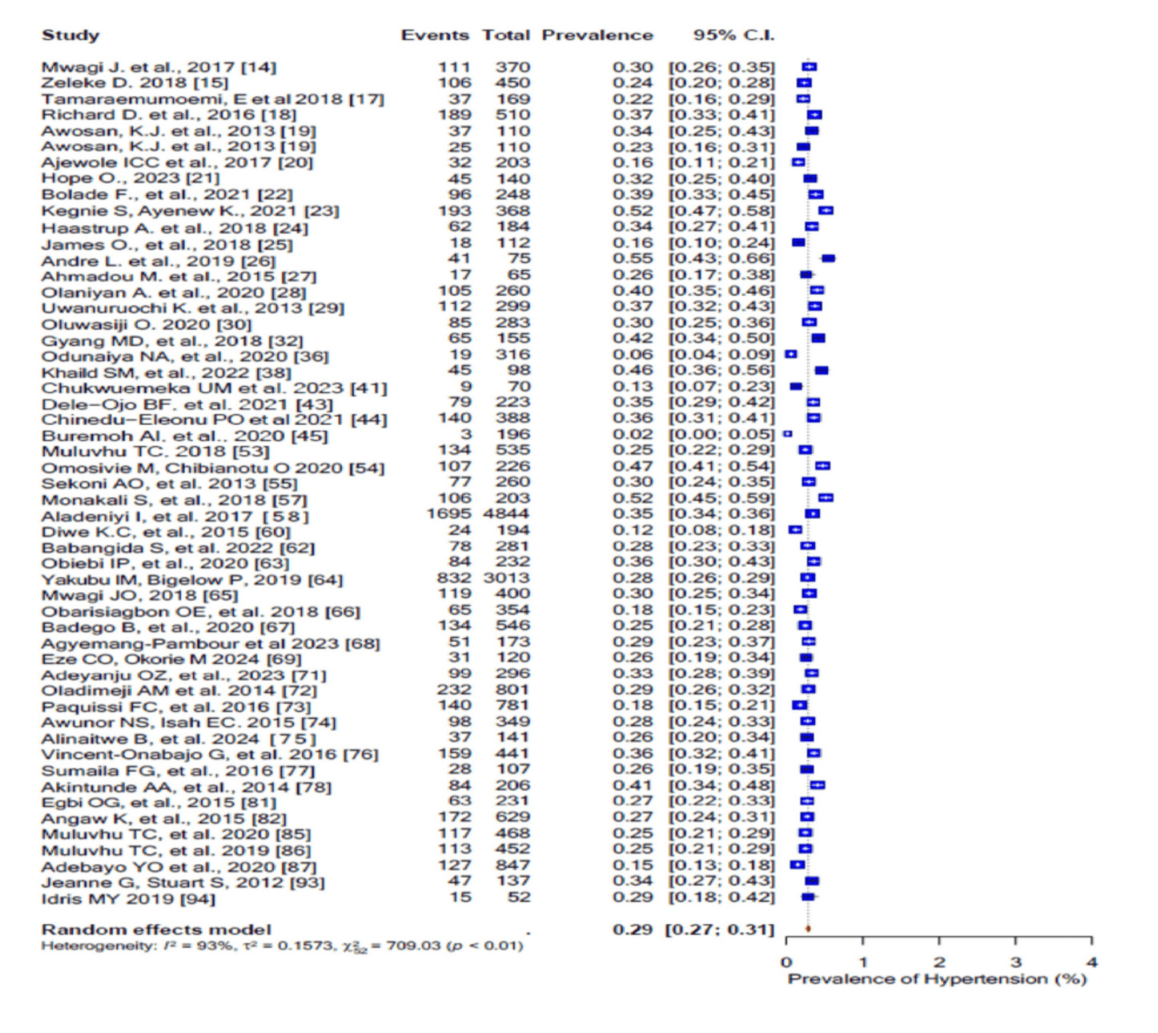
Forest plot of the prevalence of high blood pressure among the corporate workforce in Sub-Saharan Africa.

Overweight had a pooled prevalence of 36% (95% CI: 0.33-0.38) (Figure 11), obesity had a pooled prevalence of 23% (95% CI: 0.20-0.27) (Figure 12), and central obesity had a pooled prevalence of 44% (95% CI: 0.36-0.53) (Figure 13). The pooled prevalence for the history of hypertension was 16% (95% CI: 0.11-0.23; I2 = 89%), while 29% (95% CI: 0.27-0.31; I2 = 93%) of the participants had hypertension on measurement (Figure 14).

**Figure 15:**
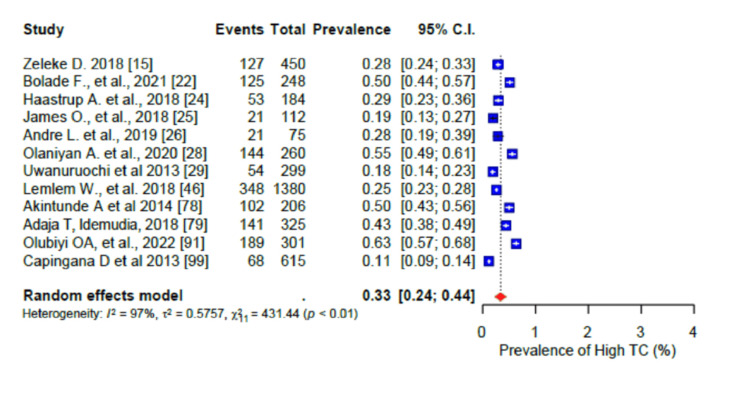
Forest plot of the prevalence of the high total cholesterol (TC) level among the corporate workforce in Sub-Saharan Africa.

**Figure 16:**
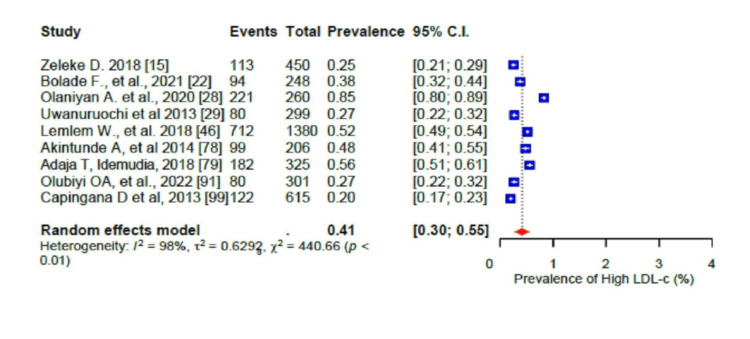
Forest plot of the prevalence of the high low-density lipoprotein (LDL) cholesterol level among the corporate workforce in Sub-Saharan Africa.

**Figure 17:**
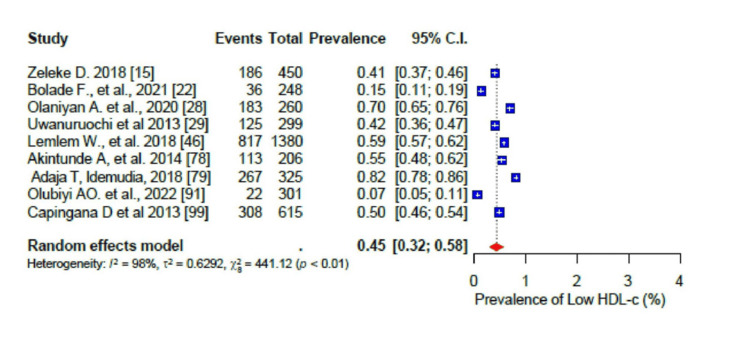
Forest plot of the prevalence of the low high-density lipoprotein (HDL) cholesterol level among the corporate workforce in Sub-Saharan Africa.

**Figure 18:**
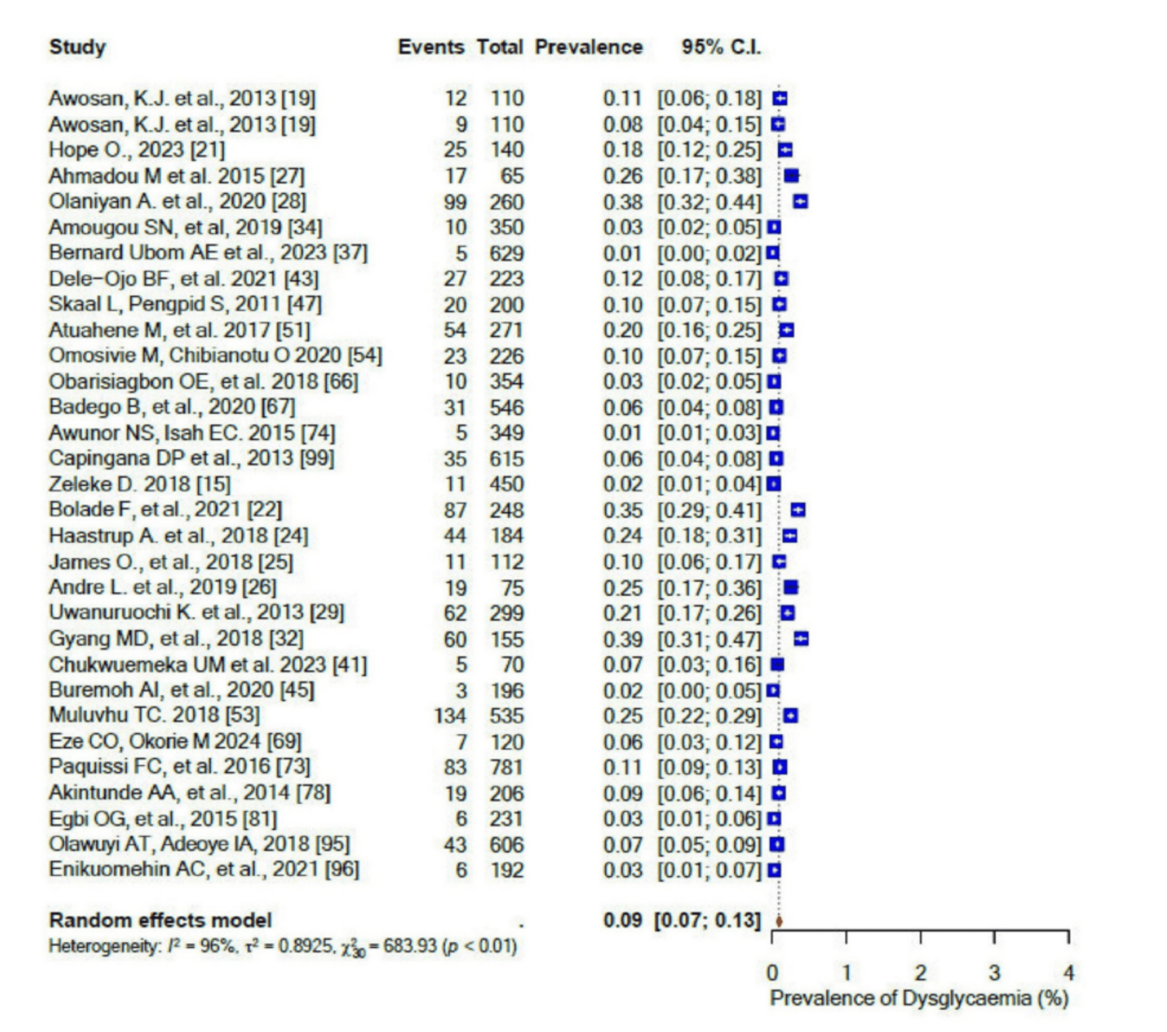
Forest plot of the prevalence of dysglycaemia among the corporate workforce in Sub-Saharan Africa.

**Figure 19:**
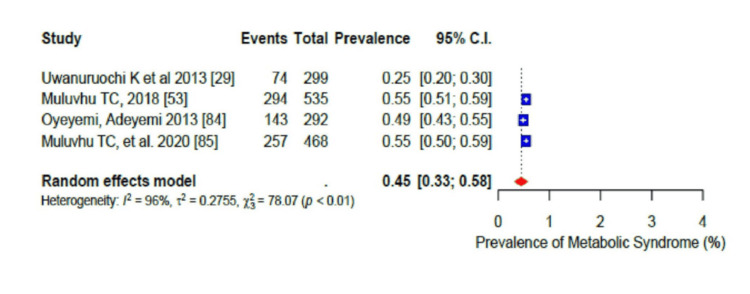
Forest plot of the prevalence of the metabolic syndrome among the corporate workforce in Sub-Saharan Africa.

For the pooled prevalence of the different components of dyslipidaemia among the participants, the pooled prevalence of high total cholesterol (TC) was 33% (95% CI: 0.24-0.44) (Figure 15), that of high low-density lipoprotein cholesterol (LDL-c) was 41% (95% CI: 0.30-0.55) (Figure 16), that of low high-density lipoprotein cholesterol (HDL-c) was 45% (95% CI: 0.32-0.58) (Figure 17), while only 17% (95% CI: 0.8-0.31; I2= 99%) had high triglycerides (TGs). In addition, the pooled prevalence of dysglycaemia was only 9% (95% CI: 0.7-0.13; I2 = 96%) (Figure 18). The pooled prevalence of MS was 45% (95% CI: 0.33-0.58) among the study participants (Figure 19). All results showed high levels of heterogeneity (I2 = 96%-98%).

Comparing the CVD risk factor prevalence before and after mitigations across the included studies

**Figure 20:**
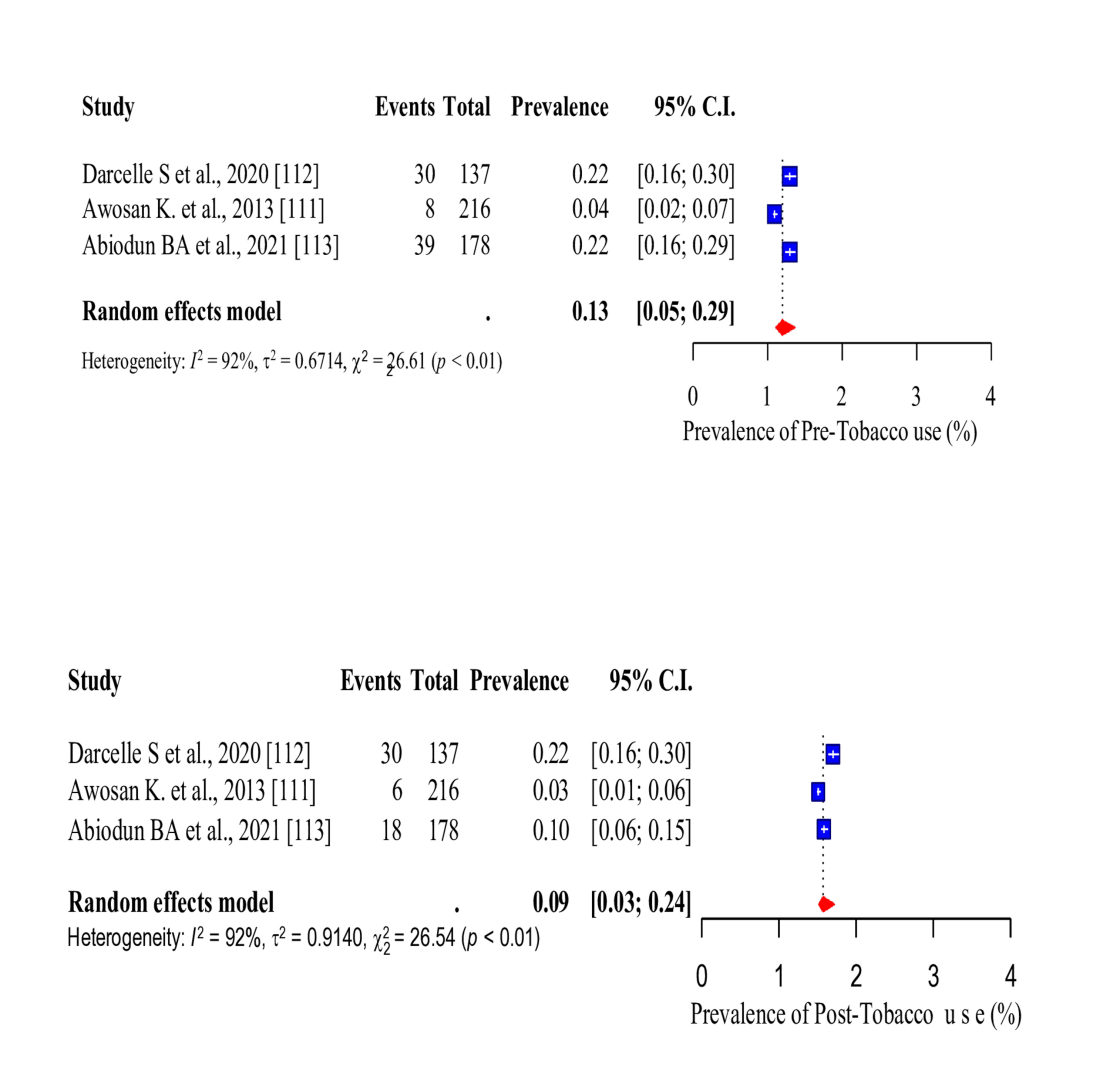
Forest plot showing the pre and post-intervention pooled prevalence for tobacco smoking.

**Figure 21:**
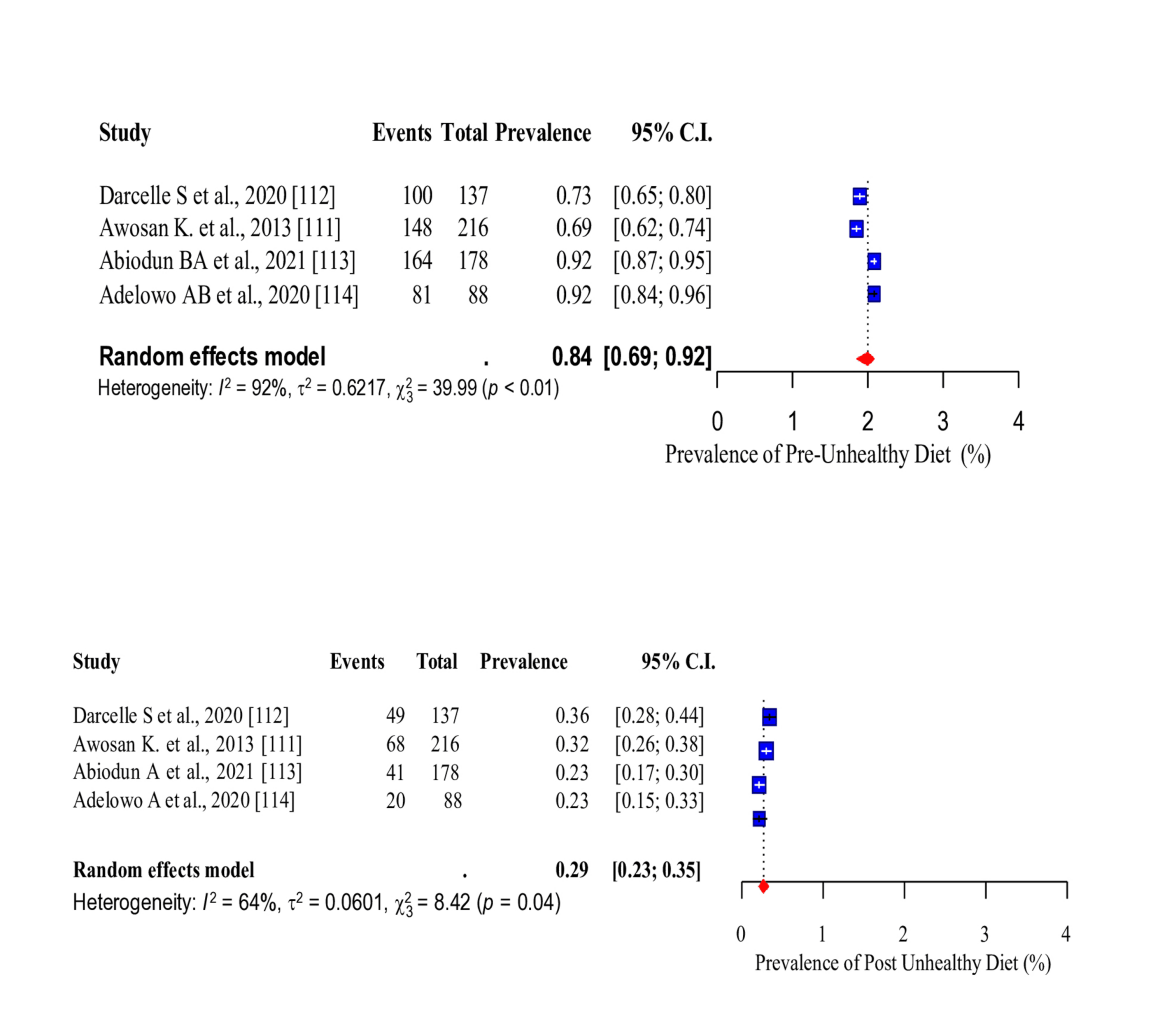
Forest plot showing the pooled prevalence of unhealthy diet before and after the intervention.

**Figure 22:**
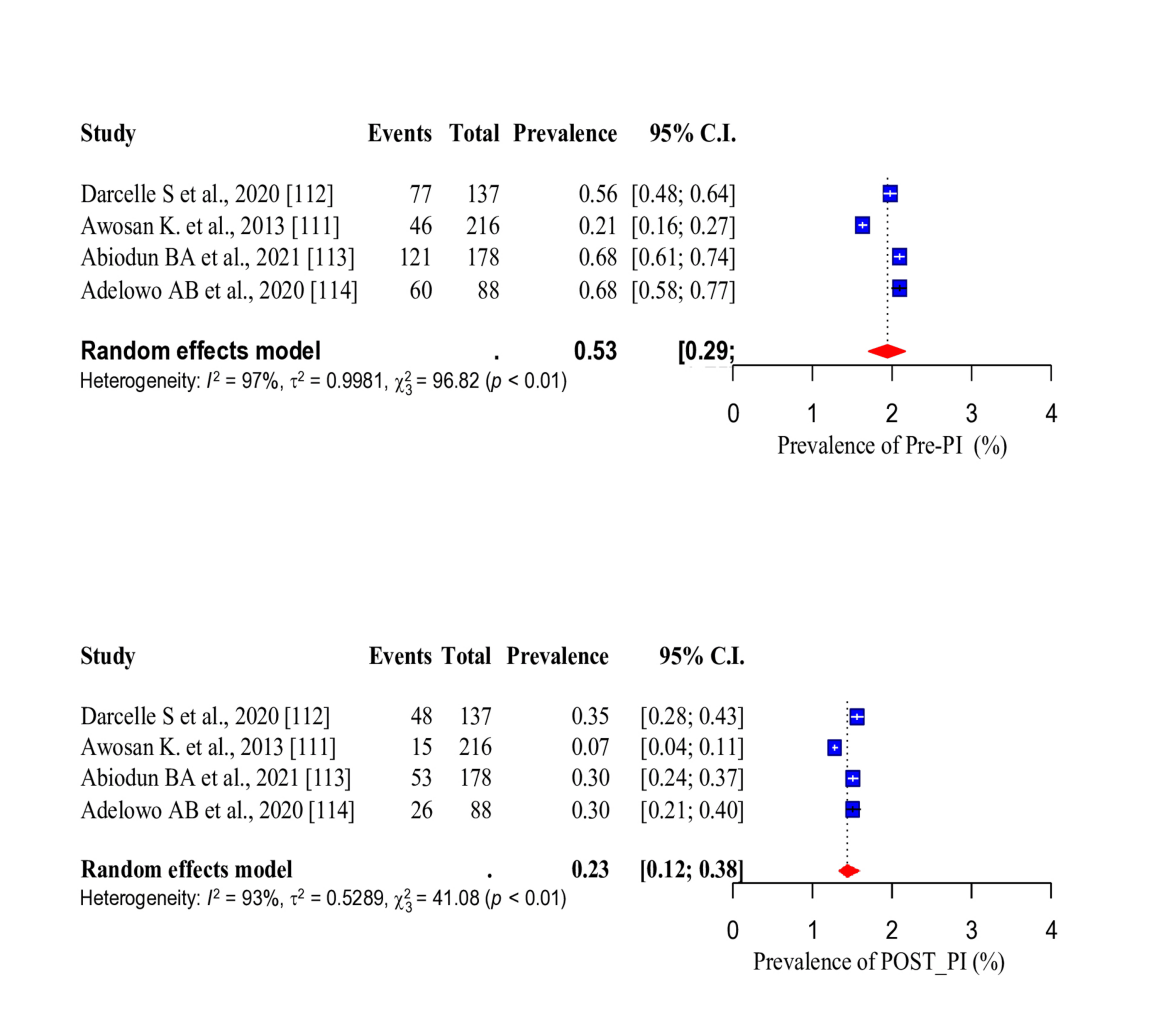
Forest plot showing the pooled prevalence of physical inactivity before and after the intervention.

Three risk factors (tobacco smoking, unhealthy diet and PI) were common in the studies that conducted WWP among the study participants. Thus, the baseline and post-intervention pooled prevalence of these risk factors were assessed. The pooled prevalence of tobacco smoking before intervention was 13% (95% CI: 0.05-0.29). After the intervention, there was a significant decrease in the pooled prevalence of tobacco smoking to 9% (95% CI: 0.03-0.24) (Figure 20). The pooled prevalence of unhealthy diet before intervention was 84% (95% CI: 0.69-0.92), which was significantly reduced to 29% (95% CI: 0.23-0.35) after the intervention (Figure 21). In addition, the pooled prevalence of PI before intervention was 53% (95% CI: 0.29-0.75), which also significantly reduced to 23% (95%CI: 0.12-0.38) after intervention (Figure 22).

Publication bias

**Figure 23:**
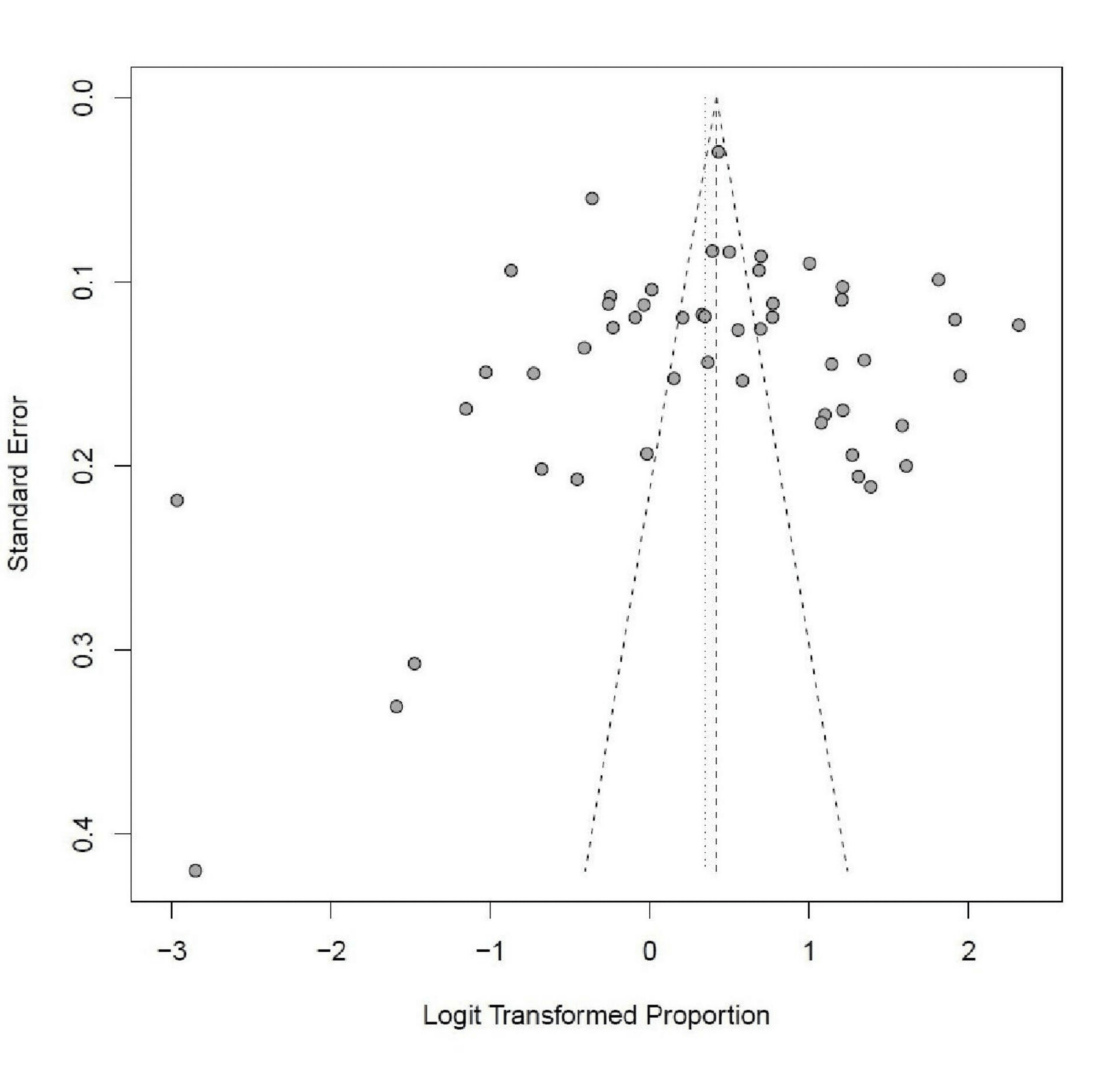
Funnel plot showing publication bias for the studies included in this meta-analysis.

The meta-analysis included a visual investigation of funnel plots to assess the presence of publication bias among the included studies. The results suggest that publication bias was unlikely to be a significant issue in the meta-analysis. Figure 23 shows the funnel plots of all assessed risk factors.

Discussion

Prevalence of Behavioral Risk Factors Among the Corporate Workforce in SSA

The pooled prevalence of unhealthy diets in this study was very high (80%). This finding is higher than the WHO estimate of 71% for the general African population that consumes less than five servings of vegetables and fruits per day [117]. This result is congruent with those of similar studies in other regions of the world. A nationwide cross-sectional study among 10,700 Spanish workers, reported that 60% and 40% do not eat vegetables and fruits, respectively, per day [118]. A recent global systematic review among oil and gas workers noted that the total fat intake of more than 50% of the participants was higher than the dietary recommendation; about half consumed more than 300 mg of cholesterol/day, and only 12.4% consumed adequate dietary fibre [119].

Another study among healthcare workers in Australia found that 85% and 49.4% of them did not meet the daily recommended servings of vegetables and fruits, respectively, and 28.5% consumed sugar-containing beverages weekly [120]. Thus, the findings of this study suggest a very high prevalence of unhealthy diet among corporate workers in SSA, higher than the general population in the region and the prevalence of unhealthy diet is also high among many workers globally.

The exact reasons for the high prevalence of unhealthy diets among the study participants are not known. However, this may be related to the presence of workplace-related barriers to healthy eating, such as time constraints to seek/prepare healthy foods, frequent exposure to unplanned (often unhealthy) food at work, negative coworker influence, eating unhealthy foods as a stress-coping strategy and non-availability of healthy dietary options/canteens in the workplace [121,122]. Thus, measures to reduce these barriers should be implemented to reduce the risk of CVD among corporate workers in SSA.

This study shows that more than half (59%) of the study participants are physically inactive. This finding is far higher than the WHO estimate of 28% of global insufficient PI levels in adults (18 years and above) [1]. It is also higher than the WHO’s estimate of 49% and 11% for insufficiently physically active and physically inactive Africans, respectively [117]. However, this result is congruent with those of many similar studies in other regions of the world. A study involving 293 Polish workers noticed the prevalence of low PI to be about 70%, 50%, and 35% among the general administrative, bankers, and civil administrative workers, respectively [123]. In addition, a recent cross-sectional study of 106 primary healthcare physicians in Saudi Arabia reported a PI prevalence of 34.8% [124]. Thus, the findings of this study suggest a high prevalence of PI among corporate workers in SSA, higher than the general population in the region, but congruent to the prevalence of PI among many other corporate workers globally.

The exact reason for the high prevalence of PI among the study participants is not known. Some potential reasons include time constraints to engage in exercise and an unsupportive workplace environment [125]. It has been estimated that approximately 77% of the waking time of most corporate workers is spent insufficiently active at work [126]. Efforts to increase physical activity in the workplace can result in enormous positive outcomes such as decreased risk and cost of treating many chronic diseases, increased productivity and return on investment, and reduced absenteeism and employee turnover [127].

Recent studies have shown that chronic stress is an important risk factor [128-130]. Even a specific stress-induced coronary syndrome, called stress (Takotsubo) cardiomyopathy or transient left ventricular apical ballooning cardiomyopathy, has been documented [128]. This study showed a high pooled prevalence of 58% for stress levels among the participants. This result is higher than the findings of a global survey that reported a 41% daily stress level among global workers, and a 46% daily stress level among the SSA workforce [131]. This result is also higher than the result of a study conducted among non-corporate workers (waiters) in Ghana, which revealed a stress level of 34.4% [132]. The result is, however, lower than the result of the 2020 workplace stress survey in the United Kingdom, which recorded 79% work-related stress among UK employees [133]. In addition, approximately 83% of workers in the United States experience daily work-related stress, while 52% suffer from burnout [134].

The findings of this study suggest a high prevalence of stress among corporate workers in SSA. Studies from other regions of the world also suggest that work-related stress is generally high among many workers globally. The high prevalence of work-related stress might be due to the presence of work-related psychosocial hazards (stressors), such as toxic work environments, unsuitable work equipment, heavy workloads, poorly defined or inappropriate work tasks or schedules, poor organisational culture, ambiguous or conflicting roles, career stagnation, lack of control over work, and poor social relationships with superiors/co-workers [135,136]. Thus, to reduce work-related stress, it is imperative to mitigate work-related hazards and increase workers’ resilience.

Sleep is a basic physiological need of humans and getting seven to nine hours of sleep most nights has been recommended for optimal physical and mental well-being [137,138]. Multiple studies have associated poor sleep with an increased risk and severity of CVD [138-141]. Sleeping for five hours or less has been associated with an approximately 200% to 300% higher risk of developing significant coronary artery calcification [138]. This study shows that more than half (59%) of the corporate workers in SSA experience poor sleep. This result is congruent with the position of the Sleep Foundation Society which stated that over 45% of people of black descent sleep less than seven hours per night [142].

However, this result is lower than that of a recent study among non-corporate workers (waiters) in Ghana, which reported a prevalence of 74% for poor sleep quality [132]. Many studies among corporate workers in other regions of the world also noticed similar findings. A recent study among employees of four companies based in Singapore noticed 42.5% of poor sleep quality, and 66.2% short sleep duration (<7 hours/night) [143]. In addition, a study conducted among 97 companies in Germany found that 49.7% of employees had moderate difficulty in initiating and/or maintaining sleep [144].

The findings of this study suggest a high percentage of the corporate workforce in SSA is experiencing regular poor sleep. Other studies from the rest of the world also suggest that poor sleep is generally high among many workers. However, the exact reasons for these results are unknown. However, it may be related to unhealthy workplace culture and practices in many organisations that increase the prevalence of the five major circadian rhythm alignment disruptors (shift working, late sleep timing, late meal timing, sleep irregularity and late chronotype) among the workforce [145,146]. Moreover, working in many corporate workplaces has been associated with insufficient sunlight exposure, which may result in vitamin D deficiency and disruption of melatonin circadian rhythm, both of which are implicated in poor sleep [147,148].

Alcohol consumption is another important risk factor for CVD [149]. Recent evidence has suggested that all amount of alcohol consumption (regardless of the quantity or type) increases the risk of developing CVD [149-151]. However, the cardiovascular system damaging effects of alcohol become more prominent when it is taken beyond the recommended amount and guidelines [1,152,153]. In this study, the pooled prevalence of current and harmful alcohol consumption was 29% and 26%, respectively. This result is lower than the WHO’s estimate of 38.3% for current alcohol drinkers globally, but congruent with the WHO estimate of 29.8% for current alcohol drinkers in the African region [154]. However, the result is way higher than the WHO’s estimate of 1.8% and 1.9% of harmful alcohol drinkers globally and in the African region, respectively [154].

The results of this study are also higher than those of other corporate workers in other regions of the world. A recent systematic review and meta-analysis that was conducted among healthcare professionals globally, involving 64 studies and 9,108 participants, reported a pooled prevalence of 20%, 3.17%, 14.6% and 17.7% for hazardous alcohol drinking, harmful drinking, dependent drinking and frequent binge drinking, respectively [155]. Another study among Italian workers noted that 16.5% engaged in unhealthy alcohol consumption, of which 8.7%, 7.3% and 4.0% engaged in binge drinking, alcohol consumption without eating and heavy alcohol consumption, respectively [156]. Another study conducted among 322 Brazilian public servants reported a prevalence of 12.7% and 32.5% for harmful drinking and binge drinking, respectively, whereas 5.3% had alcohol-related health problems [157].

The result of this study suggests that the prevalence of alcohol consumption and harmful alcohol consumption are moderately high among corporate workers in SSA. In addition, the prevalence of harmful alcohol consumption is higher among corporate workers in SSA than among the general population in Africa and many other workers in other regions of the world. Some possible explanations for these results include alcohol consumption due to peer pressure or as a negative coping strategy for the barrage of work-related challenges [158].

Tobacco use (including smokeless tobacco) is a major traditional risk factor for CVD [1,153,159-161]. Compared to nonsmokers, the risk of developing heart disease increases two to four times, and the risk of developing stroke doubles in cigarette smokers [162]. Khat is an amphetamine-like green leaf that is chewed like tobacco and can increase the risk of developing CVD [163]. In this study, the pooled prevalence of current tobacco smoking and khat chewing among the participants was 7% and 6%, respectively. The finding of this study is far lower than the WHO’s estimate of 20.9% of current tobacco users (15 years and older) globally. It is, however, close to the WHO’s estimate of 9.5% of current tobacco users (15 years and older) in Africa [164].

When compared to the workers in other regions of the world, it seems the prevalence of current tobacco use among workers is higher in many other regions of the world. The National Health Interview Survey in the United States (from 2014 to 2016) showed that 22.1% of American workers currently use tobacco products [165]. In a recent survey of 1,057 public servants in Kuwait, it was noticed that 26% of the workers used all forms of tobacco products and 20.3% smoked only cigarettes [166]. Another study conducted among employees of Lyon University Hospital France reported a prevalence of current tobacco use of 25% [167].

The result of this study suggests that the prevalence of tobacco use among corporate workers in SSA is congruent with the prevalence of tobacco use among the general population in the region. However, it is lower than the current prevalence of tobacco use among workers in other regions of the world. This result is supported by a recent 2024 WHO report which stated that, compared to other continents, Africa presently has the lowest prevalence of current tobacco use among people aged 15 years and older [164].

The exact reasons for the use of tobacco by corporate workers in SSA are unknown. Tobacco products may be used as a negative coping strategy for work-related psychosocial hazards [166]. Implementation of tobacco-free workplace policies, regular awareness/education programs, and tobacco cessation programs should help mitigate this trend [168,169].

Prevalence of intermediate risk factors among the corporate workforce in SSA

Body mass index (BMI) above the normal limit is a well-established risk factor for CVD [117,159,170,171]. At least 23% of the global burden of ischaemic heart disease can be attributed to high BMI [117]. Studies have also shown that central obesity increases the risk of CVD [172-175]. Many studies have shown that central obesity correlates better with CVD than general obesity [175,176]. In this study, the pooled prevalence rates of overweight, obesity and central obesity were 36%, 23% and 44%, respectively. The result of this study is lower than the WHO’s estimate of global overweight prevalence of 43% in adults (18 years and older), but higher than the WHO’s estimate of global obesity prevalence of 16% in adults (18 years and older) [177].

The prevalence of central obesity in this study was also slightly higher than that reported in a recent systematic review and meta-analysis (involving 13.2 million participants), which indicated a global prevalence of central obesity of 41.5% [178]. However, the same study informed that the prevalence of central obesity in Africa is 49.6% [178], which is slightly higher than the 44% noticed in this study. The result of this study is also higher than the report of the 2012 World Health Statistics which informed that 14.9% and 22.1% of African men and women respectively are obese [179].

When compared with other regions of the world, there seems to be a similar trend of high BMI among many workers. A recent systematic review and meta-analysis of nurses worldwide (involving 158,775 participants and 83 studies across 29 countries) reported the prevalence of overweight and obesity to be 31% and 16.3%, respectively [180]. Another recent study that was done among Turkish healthcare professionals noticed a prevalence of 37.8% for overweight and 11.8% for obesity [181]. A study conducted among 37,626 employees working in Washington, United States reported an obesity prevalence of 24.6% [182]. Yet another study done among 692 petrochemical workers in Iran noticed a central obesity prevalence of 27.3% [183].

The result of this study suggests that the prevalence of overweight and obesity is moderately high, while central obesity is high among the corporate workforce in SSA. Except for central obesity, the prevalence was higher than the available results in the general population of SSA and many other workers in other regions of the world. Some possible explanations for high BMI among corporate workers in SSA include lifetime exposure to an urban working environment that encourages an unhealthy diet, PI, and other obesity-related lifestyles [159]. To mitigate the high BMI among workers, the WHO has advocated for WWP in every organisation [168].

Hypertension is a major and common risk factor for CVD [159,184,185]. At least 45% of global deaths from ischaemic heart disease and 51% of global deaths from stroke can be attributed to high systolic blood pressure [117]. The pooled prevalence of hypertension among participants in this study was 45% (16% for history of hypertension and 29% for measurement).

This result is higher than the WHO report for the global population, which informed that 1 in 3 adults globally are hypertensive [186]. This result is, however, supported by the 2018 WHO report which informed that the African region has the highest prevalence of hypertension globally [1]. This result is also congruent with the estimated hypertension prevalence of 48% in SSA women, but higher than the estimated hypertension prevalence of 34% in SSA men in a study [187]. The result of this study is also higher than the report of another recent study which suggests that the prevalence of hypertension in Africa is 30.8% and between 30.0% and 31.1% in SSA [188].

The results of this study also appeared to be higher than those of many studies among workers in other regions of the world. A recent study conducted among 3,480 University Hospital employees in Turkey revealed a prevalence of 14.8% [189]. A study conducted among the Chinese workforce (involving 61 workplaces and 37,856 participants) revealed an age-standardised hypertension prevalence of 23.3% [190]. In addition, a systematic review of global healthcare workers noted that the prevalence of hypertension was between 13% and 40% [191]. Furthermore, a recent study of 692 male petrochemical workers in Iran reported a hypertension prevalence of 34.1% [183].

The results of this study suggest that the prevalence of hypertension is high among corporate workers in SSA and higher than the estimated prevalence of hypertension among the global population, the general population in SSA, and among many other workers in the world. Although the exact reasons for this outcome are not apparent, some of the suggested contributing factors include the high prevalence of unhealthy lifestyles, obesity, and low literacy levels among workers [192].

Dyslipidaemia is also an important traditional risk factor for CVD with a linear relationship with the disease condition [117,193,194]. According to the WHO, dyslipidaemia is associated with more than 50% of ischemic heart disease incidence globally, and more than 4 million mortality every year [194]. The pooled prevalence of different components of dyslipidaemia among the participants in this study was 33% for high TC, 41% for high LDL-c, 45% for low HDL-c, and 17% for high TGs. This result is higher than the WHO’s estimate of 23.1% prevalence of hypercholesterolaemia in Africa [195]. It is also higher than the result of a recent 2018 systematic review on the prevalence of dyslipidaemia in Africa, which reported a pooled prevalence of 25.5% for high TC, 28.6% for high LDL-c, and 37.4% for low HDL-c [195]. However, the pooled prevalence of high TG in the 2018 systematic review was 17% [195], which is congruent with the result of this study.

The result of this study is also far higher than the result of a recent survey that was conducted on 699 healthy Nigerians, which shows a prevalence of 5.3% for high TC, 19.3% for high LDL-c, and 4.4% for high TG, although with a higher low HDL-c (76.3%) [196]. The result from other studies in other regions of the world also suggests that the prevalence of dyslipidaemia is high among many workers. In a recent study that was done among 302 academic staff in Bangladesh, 85% of the participants were noticed to have dyslipidaemia, where high TC was 23%, high LDL-c was 24.7%, low HDL-c was 77.3% and high TG was 49.7% [197]. A study that was done among 300 male employees in India shows an overall dyslipidaemia prevalence of 50.7%, out of which high TC was 15.3%, high LDL-c was 23%, low HDL-c was 62%, high TC was 27% and high TC/HDL-c ratio was 50.7% [198].

Another study that was done among employees of a pulp and paper company in Brazil noticed a prevalence of 38.7% for high TG, 46.5% for low HDL-c, 6.5% for high LDL-c and 3.2% for mixed hyperlipidemia [199]. Yet another study that was done among 461 primary healthcare workers in Saudi Arabia noticed the prevalence of overall dyslipidemia to be 78%, where 38.7% had high TC, 43.5% had high LDL-c, 45.2% had low HDL-c and 17.4% had high TG [200]. Furthermore, another study that was conducted among 692 petrochemical workers in Iran also noticed the prevalence of the following components of dyslipidaemia among the study participants: high TC was 37.8%, low HDL-c was 71.6% and high TGs were 49.5% [183].

The result of this study suggests that the prevalence of dyslipidaemia is significantly high among corporate workers in SSA, higher than that of the general population in Africa. A generally unhealthy workplace culture and environment that encourages a sedentary lifestyle and unhealthy diet in the workplace may contribute to these observed outcomes [192].

Diabetes is a major independent risk factor for CVD [184,170,201]. It increases the risk of developing CVD by two to four times, while CVD is the highest cause of diabetes-related mortality [202]. Impaired glucose tolerance (IGT) (prediabetes) can also increase the risk of developing CVD [117,203]. Thus, dysglycaemia (IGT and diabetes) is a major contributor to the high prevalence of CVD globally. According to the WHO, high blood glucose (IGT and diabetes) is attributable to at least 22% of ischaemic heart disease and 16% of stroke deaths globally [117].

The pooled prevalence of dysglycaemia among the participants in this study was 9%, and the pooled prevalence of diabetes alone was 12.8%. This result is slightly higher than the International Diabetes Federation (IDF) estimated global diabetes prevalence of 10.5%, but significantly lower than the IDF estimated global dysglycaemia prevalence of 21.1% (where IGT is 10.6% and diabetes is 10.5%) [203]. The result is also higher than the IDF-estimated diabetes prevalence of 4.5% in Africa, but significantly lower than the IDF-estimated dysglycaemia prevalence of 17.1% (where IGT is 12.6% and diabetes is 4.5%) in Africa [203].

The result from other studies in other regions of the world also suggests that the prevalence of IGT, diabetes, and dysglycaemia is moderate to high among many workers. In a recent study that was done among 55,452 Japanese workers, the prevalence of diabetes was 3.3% in women and 8.0% in men, while the prevalence of IGT was 9.2% in women and 14.1% in men [204]. Another study conducted among 366,633 employees working in 36 States in the United States shows a diabetes prevalence of 6.4% among the participants [205]. Yet another study that was done among 16,896 South Korean workers noticed the prevalence of diabetes to be 11.6% in managers, 7.5% in clerks, 6.7% in professional workers and 6.0% in service/sales workers [206]. Moreover, a study that was done among 692 petrochemical workers in Iran noticed the prevalence of dysglycaemia to be 13.4% among the participants [183].

The results of this study suggest that although the prevalence of dysglycaemia among corporate workers in SSA may be lower than the IDF-estimated prevalence for the African region, the prevalence of diabetes is higher among the corporate workforce in SSA than in the general population in Africa. An unhealthy workplace environment may contribute to the high prevalence of dysglycaemia [201]. To address these issues, the IDF and other experts have advised that the principles and practices of a healthy lifestyle should be adopted in the workplace [126,160,170,201].

MS is a cluster of interconnected risk factors that significantly increase the risk of both CVD and type 2 diabetes mellitus [207,208]. The pooled prevalence of MS among the participants in this study is significantly high at 45%. This result is higher than the result of a recent study that was done among 325 healthy Ethiopian adults where the prevalence of MS was noticed to be 20.3% [209]. Also, a recent systematic review and meta-analysis done among Ethiopian adults noticed a pooled prevalence for MS to be 34.89% (using the NCEP/ATP III criteria) [210].

Also, a recent systematic review compared the prevalence of dyslipidaemia among hospital-based and population-based studies in Nigeria. Among the hospital-based studies, the pooled prevalence of MS was 38.4%; while in the population-based studies, the pooled prevalence of MS was 18.3%, and the mean overall prevalence of MS was 27.9% (using the ATPIII criteria) [211]. Moreover, a recent study done among 692 male petrochemical workers in Iran noticed an MS prevalence of 15.1% [183]. Thus, the results of this study suggest that the prevalence of MS is significantly higher among corporate workers in SSA than the available prevalence of MS among the general population in SSA and some workers in other regions of the world.

Hwang and Kang suggested that compared to the general population, workers are at higher risk of developing MS due to the heavy workload and high prevalence of unhealthy lifestyles that are common in many workplaces [136]. Thus, a comprehensive workplace intervention that emphasises healthy habits and addresses the individual components of MS and other CVD risk factors is needed to reduce the risk of developing CVD among SSA workers [212].

Comparing the CVD risk factor prevalence before and after mitigations across the included studies

Out of the included studies, only 6 (5.7%) conducted WWPs/health promotion programmes or any form of intervention to mitigate the identified CVD risk factors among the corporate workers in SSA. The intervention period ranges from six weeks [115] to two years [112]. The components of the interventions in the various studies included Risk Appraisal (six studies), oral health education sessions (five studies), health education manuals (three studies), exercise programs (four studies), healthy diet programs (two studies), behavioural change counselling sessions (two studies), social media follow-up support (four studies) and top management motivational messages (one study).

The pooled prevalence of three risk factors (tobacco smoking, unhealthy diet and PI) that were common to most of the interventions was analysed. The pre-intervention pooled prevalence of tobacco smoking, unhealthy diet and PI was 13%, 84% and 53%, respectively. After the interventions, there was a significant decrease in post-intervention pooled prevalence of 9%, 29% and 23%.

Many other studies that have conducted workplace wellness interventions in different regions of the world have reported positive outcomes. A recent systematic review and meta-analysis (involving 33 systematic reviews and 24 meta-analyses) revealed that WWP significantly reduced participants’ body weight (16 studies with a mean difference of -2.61 kg), waist circumference (13 studies with a mean difference of -1.92 cm), and BMI (19 studies with a mean difference of -0.42 kg/m2) [126]. Another systematic review involving 52 RCTs, 2 randomised crossover design studies, 31 non-randomised pre-post control group studies, and 51 non-randomised pre-post non-control studies noticed that after WWP, there was a significant improvement in dietary habits (8/9), physical activity level (11/14), indices of body composition (20/24) and stress level (49/66) [213].

Furthermore, another intervention study conducted among 719 Ferrari car manufacturing workers in Italy revealed that WWP significantly improved the workers’ cardiorespiratory fitness profile (estimated V02, METs, Watt) and many CVD risk factors, such as BMI, blood pressure, TC, LDL-c and TGs [214]. Yet another systematic review and meta-analysis (involving 82 RCTs and 39 quasi-experimental studies) across different regions of the world, noticed that after WWPs, there was a significant improvement in fruit consumption only (0.20 servings/day), combined fruit & vegetable consumption (0.27 servings/day), total fat intake (-1.18% of daily energy intake), saturated fat intake (-0.70% of daily energy), body weight (-0.92 kg), waist circumference (-1.47 cm), BMI (-0.22 kg/m²), systolic blood pressure (-2.03 mmHg), diastolic blood pressure (-1.11 mmHg), fasting blood glucose (-1.81 mg/dL) and different components of dyslipidaemia (LDL-c by -5.18 mg/dL, HDL-c by 1.11 mg/dL, and TGs by -5.38 mg/dL) [215].

The results of this systematic review and meta-analysis suggest that an evidence-based multicomponent WWP can significantly reduce multiple CVD risk factors among corporate workers in SSA. The results from similar studies in different regions of the world also corroborated these findings.

Limitations

The varying definitions and screening criteria used by many studies assessing some of the CVD risk factors are a limitation. The most commonly observed are the varying definitions and screening for unhealthy diet, PI, stress and poor sleep. Furthermore, some of the studies did not specify the number of times they measured the blood pressure of their study participants before arriving at a diagnosis of hypertension.

## Conclusions

This study demonstrated that the prevalence of most CVD risk factors is high among many corporate workers in SSA, which is higher than that in the general population in most cases. The prevalence of most CVD risk factors is high among many corporate workers in other regions of the world. However, there is a significant variation in the prevalence and distribution of these risk factors in different regions, countries and work sectors of SSA. Furthermore, despite evidence to support the efficacy of multi-component WWP, only 5.7% of the included studies implemented WWP to mitigate CVD risk factors among their participants, which resulted in positive outcomes for most risk factors.

Thus, to mitigate the current CVD epidemics in SSA, community-based screening efforts for CVD risk factors need to be intensified among healthy workers, in addition to hospital-based screening measures. In addition, to reduce the high and possibly increasing prevalence of most CVD risk factors among the corporate workforce in SSA, it may be expedient for all stakeholders (government, employers and employees) to consider conducting regular evidence-based WWP among the workforce in all work sectors. To ensure success, these interventions should be delivered and monitored by individuals trained in the design and implementation of evidence-based WWP. In addition, more studies are needed to identify the specific contributing factors to this high prevalence and variation in CVD risk factors among the corporate workforce in SSA. In addition, the direct and indirect cost implications of the high prevalence of most CVD risk factors on the productivity and ROI of most corporate organisations and countries should be investigated in future studies.
